# Network analysis of pig movements: Loyalty patterns and contact chains of different holding types in Denmark

**DOI:** 10.1371/journal.pone.0179915

**Published:** 2017-06-29

**Authors:** Jana Schulz, Anette Boklund, Tariq H. B. Halasa, Nils Toft, Hartmut H. K. Lentz

**Affiliations:** 1 Technical University of Denmark, National Veterinary Institute, Kgs. Lyngby, Denmark; 2 Friedrich-Loeffler-Institut, Federal Research Institute for Animal Health, Institute of Epidemiology, Greifswald, Insel Riems, Germany; University of Minnesota College of Veterinary Medicine, UNITED STATES

## Abstract

Understanding animal movements is an important factor for the development of meaningful surveillance and control programs, but also for the development of disease spread models. We analysed the Danish pig movement network using static and temporal network analysis tools to provide deeper insight in the connection between holdings dealing with pigs, such as breeding and multiplier herds, production herds, slaughterhouses or traders. Pig movements, which occurred between 1^st^ January 2006 and 31^st^ December 2015 in Denmark, were summarized to investigate temporal trends such as the number of active holdings, the number of registered movements and the number of pigs moved. To identify holdings and holding types with potentially higher risk for introduction or spread of diseases via pig movements, we determined loyalty patterns, annual network components and contact chains for the 24 registered holding types. The total number of active holdings as well as the number of pig movements decreased during the study period while the holding sizes increased. Around 60–90% of connections between two pig holdings were present in two consecutive years and around one third of the connections persisted within the considered time period. Weaner herds showed the highest level of in-loyalty, whereas we observed an intermediate level of in-loyalty for all breeding sites and for production herds. Boar stations, production herds and trade herds showed a high level of out-loyalty. Production herds constituted the highest proportion of holdings in the largest strongly connected component. All production sites showed low levels of in-going contact chains and we observed a high level of out-going contact chain for breeding and multiplier herds. Except for livestock auctions, all transit sites also showed low levels of out-going contact chains. Our results reflect the pyramidal structure of the underlying network. Based on the considered disease, the time frame for the calculation of network measurements needs to be adapted. Using these adapted values for loyalty and contact chains might help to identify holdings with high potential of spreading diseases and thus limit the outbreak size or support control or eradication of the considered pathogen.

## Introduction

The movement of pigs between holdings is an important route of transmission for pathogens [1,2]. Therefore, trade restrictions are implemented in case of an outbreak of any highly contagious disease, e.g. foot and mouth disease and classical swine fever [3]. However, other pathogens such as livestock associated methicillin-resistant *Staphylococcus aureus* (LA-MRSA) do not invoke movement restrictions. Hence, these pathogens might spread freely via pig movements.

Facilities such as production herds, slaughterhouses and traders form a complex network. During the last decade, methods of network analyses were described and introduced into veterinary science [4,5,6,7]. Network analysis helps characterising the contacts between holdings and leads to a better understanding of the potential risk for the spread of pathogens through the production chain. Exploring livestock movements can contribute towards risk-based control strategies to prevent and monitor the introduction and the spread of infectious diseases in animal populations.

Since the EU regulations require identification and registration of pigs [8], data on pig transports are available on a large scale. Network analyses of pig movements were performed in several countries and highlighted the potential for pathogen spread and implications for control programs by estimating the potential transmission pathways between holdings connected by direct or indirect contacts [9–14].

**Fig 1:**
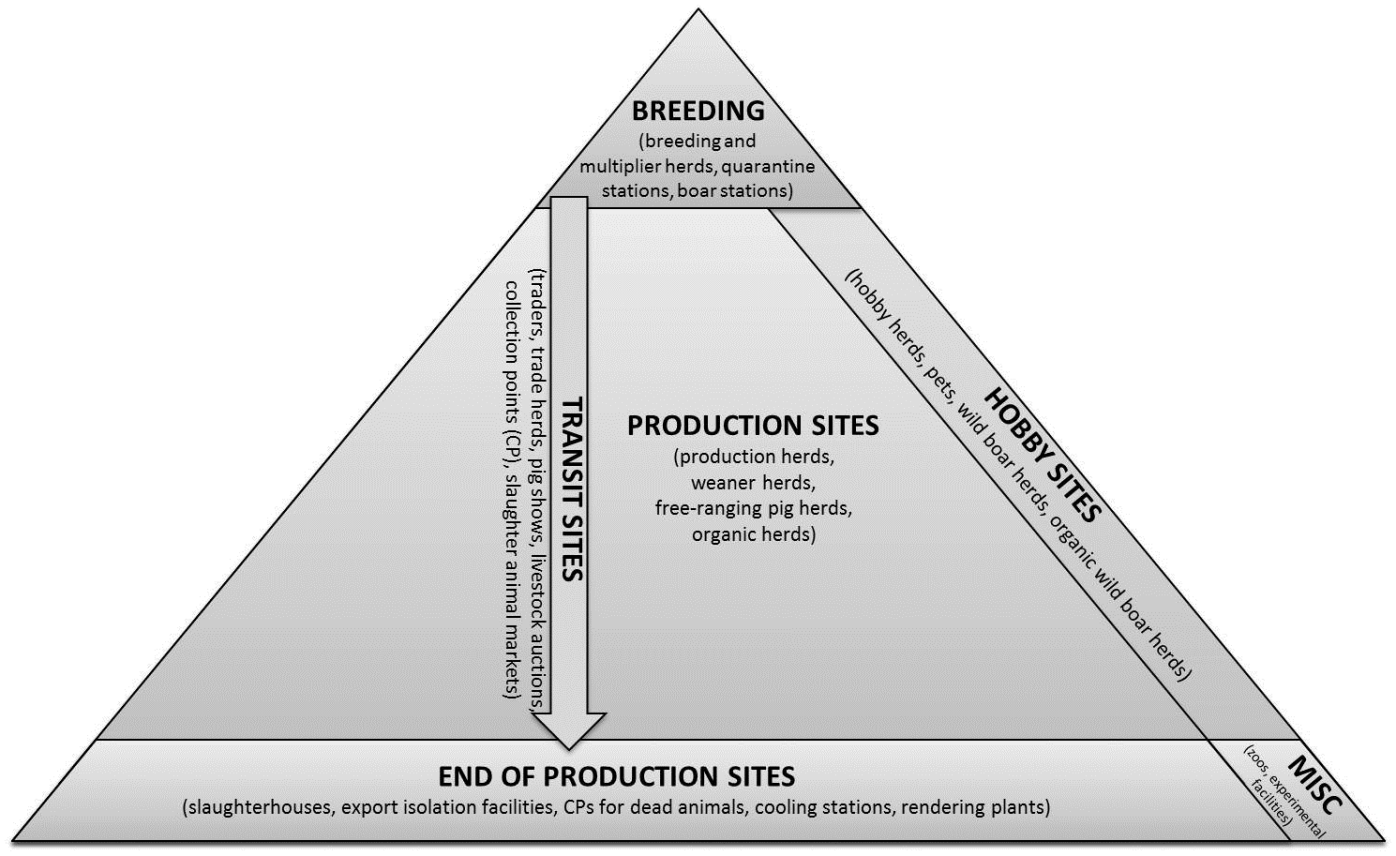
Pyramidal structure of the Danish pig production chain. The 24 holding types considered in this study were assigned to generic production steps. Besides the vertical connections, illustrated by the vertical transit sites horizontal connections also exist.

The Danish pig industry is among the leading pig industries in the world in areas such as breeding, quality, food safety and traceability. As a result, the Danish pig industry constitutes an essential part of the Danish economy, as approximately 85% of Danish pork and 13 million pigs are exported every year [15]. The Danish pig production is pyramidal structured with breeding sites on the top, production sites in the centre, down to end of production sites like slaughterhouses in the bottom of the pyramid [16] (Fig 1).

In Denmark, Bigras-Poulin et al. [16] described trade patterns of Danish pigs between Sept 2002 and May 2003. Their analysis covered only a short time period and thus actual temporal trends for the development of the pig production sector could not be gained. Nevertheless, long term analysis is essential to understand the dynamics of disease spread in complex networks such as the pig movement network in Denmark [16]. With this information the consequences of the introduction of a contagious disease can be estimated and control measures can be planned. If holdings differ from one another with respect to their potential to spread diseases, this variability can be used to rank the holdings. Such a ranking allows veterinary authorities to select holdings or holding types for the implementation of targeted surveillance and control measures.

In this study, our main goal was to provide a comprehensive description and exploration of changes over time in the structure of the Danish pig movement network in the period from 1^st^ January 2006 to 31^st^ December 2015 by use of recently developed approaches of static and temporal network analysis tools. Descriptive statistics of parameters such as the number of active holdings, the number of registered pig movements and the number of pigs moved between different types of pig holdings are presented. To identify holdings and holding types with potentially higher risk for introduction or spread of diseases via pig movements, we calculated the holdings in- and out-loyalty as a local measure of its tendency to maintain contacts with the same holdings over time. Furthermore, we extracted network components to identify subsets of holdings where connectedness is particularly high and the holding types most prevalent within connected components. Finally, we evaluated the size of the in-going and out-going contact chain for each holding by tracing back and forward all direct and indirect pig movement contacts within yearly snap shots. These investigations could be used to develop meaningful surveillance and control programs, by focussing on high risk holdings or a holding type for surveillance or monitoring. Additionally, results gained by network analysis could be used for the development of disease spread models for the simulation of movement patterns in case of missing real data.

## Material and methods

### Data set

In Denmark, information on pig movements is part of the Central Husbandry Register (CHR) [17,18]. This central database was established in 1992 and is owned by the Ministry of Environment and Food. Data from 1^st^ January 2006 to 31^st^ December 2015 representing all registered pig holdings and pig transports between holdings in Denmark were used in this study. The basis for our analysis consisted of 18,648 holdings registered in the CHR within the considered time period. The CHR data provided among others, holding and enterprise identification number, information on holding type and the number of sows, finishers and weaners in each holding. The CHR data were available on yearly basis, and thus changes in holding types and the number of pigs in each holding were accessible. There are 24 pig holding types predefined in the CHR, and the owner has to indicate the type of holding during the registration process (Fig 1). No definitions of the types of holdings are available in the CHR. In Denmark, several holdings of different types can be owned by one person and thus constitute an agricultural enterprise (farm). We performed all analyses at the holding level.

The movement data file contained 7,678,851 movement records. For each pig movement data record, sending and receiving holding and enterprise identification numbers, the number of moved pigs and the date of the pig movement are recorded. Movements among holdings owned by the same farmer are recorded and thus included in the study. As holdings within each farm can be of different types, e.g. production herd and cooling station, both holding and enterprise identification numbers were used in the analysis. From this point, we define “holding” as identified by the combination of holding and enterprise number in the CHR.

Imports to and exports out of Denmark were not included in the movement dataset. In total, 8,949 movements were excluded, because the sending (7,972) or receiving (977) holding was not registered as a pig holding but kept other species. One holding and 51,820 corresponding movements were excluded from the analysis due to mistakes during the registration of movements. Additionally, 401 out-going movements from slaughterhouses were excluded, because the receiving holding type was not a slaughterhouse or rendering plant. On follow-up investigations of these excluded out-going movements, many turned out to be registration errors. As movements out of slaughterhouses to production herds are not allowed to occur, these movements were excluded. Movements between slaughterhouses occur in case of a lack of capacity in the sending slaughterhouse. Also movements of dead animals to e.g. rendering plants are recorded in the movement database. For the sake of a complete description of the Danish pig movement network, we kept these records. We only included holdings that were at least once registered as sender or receiver of pigs, leading to 16,069 holdings and 7,617,681 movements of pigs included in the analysis.

### Data analysis

For 5,147 holdings, the type of holding was not registered in the data set. These holdings were checked manually at the CHR website and if active on 4 Jul 2016 the holding type at that date was used in the analysis. For 2006, the number of weaners was not available in the CHR. We therefore estimated the number of weaners by multiplying the given number of sows with 4.5 based on production results in the swine industry [19]. The size of the holdings was calculated as the sum of the registered number of sows, finishers and weaners. If no sows, finishers and weaners were registered, the total holding size was set to “not available”.

#### Data summaries

Holdings were assumed to be active within a year, if the holding was involved in at least one pig movement. We determined the annual number of active holdings and the annual number of registered pig movements in order to describe the changes over time. Additionally, we calculated (1) the number of holdings per type over the years, (2) the median number of pigs moved each year by type of holding for both the sending and receiving holding, and (3) the median number of pig movements per holding type, again for both the sending and receiving holding.

To show the connection between holdings of different holding types, we generated heat maps showing (1) the number of movements occurring between different holding types and (2) the number of pigs moved between two different holding types for the whole time period and yearly snapshots.

#### Static network analysis

A network was generated using the pig holdings as set of nodes *V* that are connected by pig movements as set of links *E*. Thus a link exists between two premises, if at least one pig movement was recorded in the whole time period from 2006 to 2015. The network is directed, meaning that a holding could be linked one-sided (if the holding only sends or receives pigs from another holding) or in both directions (if the holding sends and receives pigs from another holding). Additionally, yearly snapshots containing only active pig holdings and movements occurring in the considered year were generated to analyse the network over time.

To describe the level of variation of the network on a yearly basis, we calculated the network loyalty defined as the fraction of common directed links for all considered years [20]. Therefore, we calculated a yearly adjacency matrix *A*^*t*^. For a network with a set of nodes *V*, the adjacency matrix *A*^*t*^ is a square |*V*| *x* |*V*| matrix with $A_{ij}^{t}$ is one when there is an directed link from holding *i* to holding *j* in year *t*, and zero when there is no link in year *t*. The network loyalty $\Lambda_{t_{1},t_{2}}$ is then defined as the count on how often $A_{ij}^{t_{1}}=\;A_{ij}^{t_{2}}=1$ divided by $\left|V^{t_{1}}\right|$. The memory in link occurrence can be quantified using network loyalty for all possible time differences. More specifically, we define the link memory of a network as the average number of common links between all year-pairs (*t*_1_,*t*_2_), where *t*_1_ < *t*_2_ and where the memory length is measured in terms of *τ* = *t*_2_ –*t*_1_.

Node loyalty *θ* measures the fraction of preserved direct contacts (neighbours) of a holding between two consecutive years, *t* − 1 and *t*. [20]. In order to quantify $\theta_{i}^{t-1,t}$, we define $\Upsilon_{i}^{t-1}$ as the set of in- and out-going neighbours of holding *i* in year *t* − 1. Then $\theta_{i}^{t-1,t}$ is given by the Jaccard Index

$$\theta_{i}^{t-1,t}=\frac{\left|\Upsilon_{i}^{t-1}\cap \Upsilon_{i}^{t}\right|}{\left|\Upsilon_{i}^{t-1}\cup \Upsilon_{i}^{t}\right|}$$

We expanded this concept to in- and out-loyalty which measures the fractions of preserved direct neighbours (in-loyalty ^*in*^*θ*) from which a holding receives pigs (Υ init−1) or the fraction of preserved direct neighbours (out-loyalty ^*out*^*θ*) to which a holding sends pigs (Υ outit−1). Both, in- and out-loyalty were determined for all holdings in the network, the yearly snapshots and for each type of holding to compare holding types. Therefore, we aggregated the yearly in- and out-loyalty values for each holding type

θhtin =∪t=2007i ϵ Vkt2015θ init−1,t   and   θhtout =∪t=2007i ϵ Vkt2015θ outit−1,t,

provided that $V_{\;}{}_{k}^{t}$ is defined as a subset of all holdings at time *t ϵ* 2007, … 2015 of the considered type of holding *k*. To categorize the holding types, three levels for in- and out-loyalty θhtin and θhtout were used. If the average loyalty of all holdings of holding type *ht* was below 0.45 (between 0.45 and 0.55, or above 0.55), the holding type was categorized as holding type with low (intermediate, or high) loyalty.

To characterize the network, we computed the fragmentation *F*, which measures the number of paths in different components over the number of all possible paths in the network. A connected component is defined as subset of nodes *C* ⊆ *V* for which a path exists between any pair of nodes in *C*. A path between two nodes is a direct or indirect connection between them. The fragmentation is calculated as

$$F=1-\;\frac{\sum_{k}S_{k}\left(S_{k}-1\right)}{n\left(n-1\right)},$$

with *S*_*k*_ the number of holdings in component *k* and *n* the total number of holdings in the network. The fragmentation ranges between 0 and 1. A value close to 0 indicates a very connected network and a value of 1 means that every node is isolated [21].

A network component analysis was performed for the whole static network and for the yearly snapshots to identify subsets of nodes in the networks where connectedness is particularly high [21]. The general component structure of directed networks was investigated by Dorogovtsev et al. [22]. The giant strongly connected component (GSCC) is the largest subset of holdings where there exists a path between all pairs of holdings in that subset. It is a particular feature of directed networks that the existence of a giant component induces the existence of other groups of holdings. These are (1) the giant in-component (GIC), which consists of all holdings with out-going contacts to the GSCC that they are not part of, and (2) the giant out-component (GOC), which consists of all holdings receiving pigs directly or indirectly from the GSCC that they are not part of [12,22]. Additionally, we determined the holding types within the three types of components.

#### Temporal network analysis

In contrast to the static situation, the time when contacts between holdings occur and especially the chronological order of contacts is taken into account in temporal networks.

We calculated the size of the in-going and out-going contact chain for each holding, by tracing back and forward all direct and indirect contacts within the yearly snap shots [20, 21]. Both measurements could be useful when setting up strategies for disease control as they identify holdings with many contacts through pig movements and that thus are at potentially higher risk for introduction or spread of diseases.

### Software

Data processing and network analysis was done in R version 3.2.2 (R Development Core Team, 2015)—"Fire Safety" using the packages igraph [23] and epiContactTrace [24].

## Results

### Data analysis

#### Data summaries

In total, there were 24 different types of holdings in the dataset. Of the 12,814 holdings in 2006 to 7,835 holdings in 2015, 91.6to 79.5% were registered as production herds (Table 1). The total number of active holdings decreased over the considered time period. The number of active production herds also decreased, whereas the number of other holding types such as breeding and multiplier herds and boar stations remained constant. The number of weaner herds and hobby herds increased over time (Table 1).

**Table 1:** Number of active Danish pig holdings.

	2006	2007	2008	2009	2010	2011	2012	2013	2014	2015
**Breeding sites**										
Breeding and multiplier herds	278	261	252	263	270	276	272	271	260	234
Quarantine stations	71	75	62	57	48	39	46	41	36	44
Boar stations	18	17	16	15	16	16	17	17	17	17
**Production sites**										
Production herds	11,733	10,867	9,902	8,691	7,940	7,538	7,090	6,819	6,623	6,230
Weaner herds	110	105	106	171	241	240	227	224	223	201
Free-ranging pig herds	325	279	189	190	157	145	141	153	169	164
Organic pig herds	76	79	117	106	99	93	90	87	90	88
**Hobby sites**										
Hobby herds	69	101	110	196	358	391	454	561	721	521
Pets	5	2	1	1	2	2	1	3	10	11
Wild boar herds		2	1	1	1	1	15	8	8	11
Organic wild boar herds										1
**Transit sites**										
Traders	7	6	10	8	8	5	6	7	8	3
Trade herds		1	1	7	20	17	17	16	15	16
Pig shows	9	8	8	6	6	6	9	9	6	7
Livestock auctions	1	1								
Collection points (CP)	7	11	15	16	17	17	20	21	24	25
Slaughter animal markets	1	2	2					1	1	1
**Miscellaneous**										
Zoos						1	1	4	4	4
Experimental facilities			1	1	3	3	2	1	2	5
**End of production sites**										
Slaughterhouses	100	97	89	83	80	78	74	75	77	76
Export isolation facilities						3	3	3	3	3
CPs for dead animals	2	4	2	2	2	2	7	18	35	121
Cooling stations	1	1	1	1	1	1	9	30	33	51
Rendering plants	1	1	1	1	1	1	1	1	1	1
**Total**	12,814	11,920	10,886	9,816	9,270	8,875	8,502	8,370	8,366	7,835

Number of active Danish pig holdings (sent or received pigs at least once) from 1^st^ January 2006 to 31^st^ December 2015 in Denmark.

**Fig 2:**
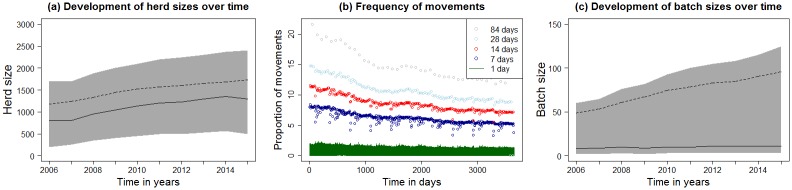
Descriptive statistics of the examined data. Figure (a) and (c) show the median (solid black line) and average (dashed black line) of holding sizes and of the number of pigs moved per pig movement sizes over time. Grey areas represent the range between 1^st^ and 3^rd^ quantile. Temporal trends of holding sizes for the investigated 24 holding types are shown in S1 File. Figure (b) shows the proportion of registered pig movements out of all possible movements in Denmark between 1^st^ January 2006 and 31^st^ December 2015 on daily base (green) and aggregated for 7 (dark blue), 14 (red), 28 (light blue) and 84 (grey) days.

The median holding size increased between 2006 and 2015 (Fig 2, S1 File). The number of holdings in the category “holding size not available” was not equally distributed over time and ranged between 3% in 2008 and 15% in 2014. Breeding and multiplier herds, production herds and weaner herds were the holding types with the highest median holding sizes (S1 File).

The overall frequency of pig movements decreased over time (Fig 2) at all levels of aggregation. Fewer movements are recorded on weekends and bank holidays. The weekly aggregated movements (7 days) showed occasional weeks with fewer movement frequencies reoccurring each year due to holidays. When looking at yearly pig movements by type of holding, the results showed: (1) a constant average number of movements were recorded out of breeding and multiplier herds and production herds, (2) a reduction of the average number of movements to slaughterhouses and rendering plants, (3) a reduction of the movements to transit sites, and (4) an increase in movements from weaner herds (S2 File).

The median number of pigs moved in one movement (batch) increased in the period between 2006 and 2015 (Fig 2, S2 File). The annual values for the maximum batch sizes were very high (mean = 8,057, ranging from 6,918 to 9,780).

**Fig 3:**
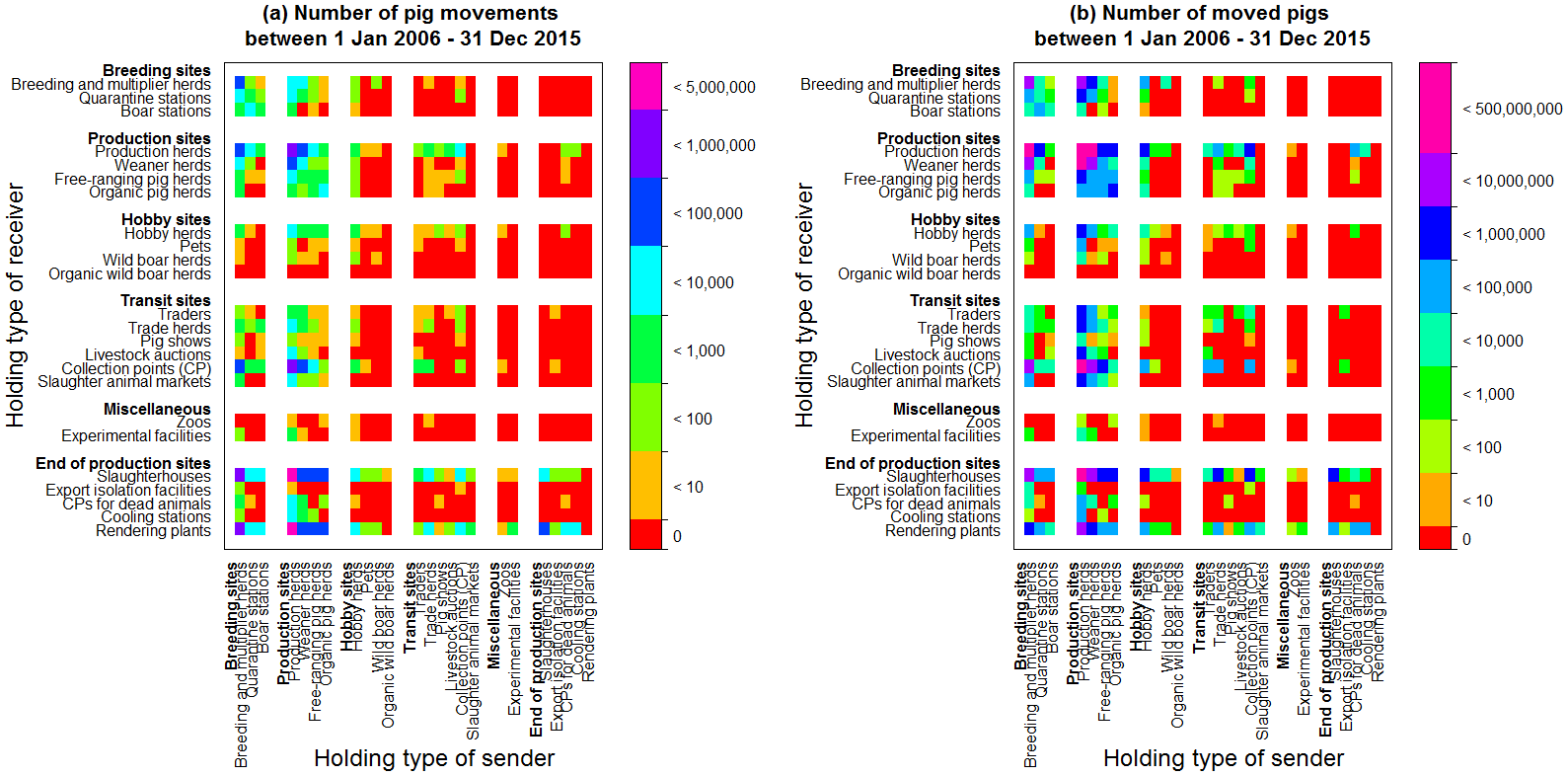
Registered pig movements and number of pigs moved. Heat maps describing (a) the number of registered pig movements and (b) the number of pigs moved between holding types from 1^st^ January 2006 and 31^st^ December 2015 in Denmark. The heat maps show stability over time and for the number of movements (data not shown).

Transports from production herds to slaughterhouses and rendering plants represented 77% of the registered pig movements (Fig 3). Looking at the number of pigs moved between two holding types, 78% of the pigs were moved from production herds to (1) production herds, and (2) slaughterhouses (Fig 3). Additionally, 15% of the pigs were moved from breeding and multiplier herds to production herds, between production herds and weaner herds and from production herds to collections points. These movements reflect the pyramidal structure of the pig production sector.

#### Static network analysis

**Fig 4:**
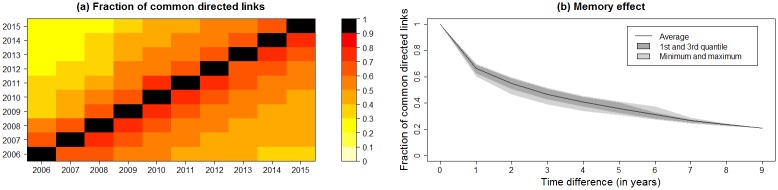
Fraction of common directed links. (a) Network loyalty (fraction of common directed links contained in two consecutive snapshots) of the pig movement network in Denmark between 1^st^ January 2006 and 31^st^ December 2015. Asymmetry is caused by varying number of directed links per year. (b) Development of the fraction of common directed links per year time difference. Dark grey areas represent the interval between 1^st^ and 3^rd^ quantile, light grey areas represent the interval between minimum and maximum values for the fraction of common directed links.

Around 60–90% of connections between two pig holdings were present in two consecutive years and around one third of the connections persisted the full period from 1^st^ January 2006 to 31^st^ December 2015 (Fig 4). Fig 4b shows the memory of common links between pairs of consecutive years. For short time differences, the number of common directed links decreases faster compared to longer time differences.

**Fig 5:**
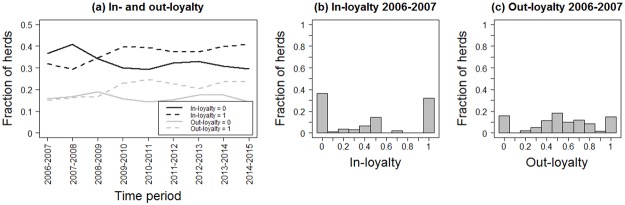
In- and out-loyalty patterns. (a) Fraction of holdings with in-loyalty and out-loyalty equal to 0 and 1 for the whole network of pig movements from 1^st^ January 2006 to 31^st^ December 2015 in Denmark. (b) and (c) show the histograms of in- and out-loyalty for the two consecutive years 2006 and 2007. Both histograms refer to the years 2006 and 2007 for visualization purposes, all other distributions show stability over time and are shown in in S2 File.

Comparing the values for node loyalty for each pair of consecutive years over time, we observed a slight shift to higher in- and out-loyalty over time (Fig 5, S3 File). In general, there were more holdings with in-loyalty equal to 1 compared to out-loyalty equal to 1. Table 2 summarizes the in- and out-loyalty per holding types (see also S3 File). Weaner herds showed the highest level of in-loyalty, whereas we observed an intermediate level of in-loyalty for all breeding sites and production herds (S3 File). Boar stations and production herds showed a high level of out-loyalty.

**Table 2:** Levels of loyalty and contact chains for different pig holding types in Denmark, based on data from the CHR, 1^st^ January 2006 – 31^st^ December 2015.

	In-loyalty	In-going contact chain	Out-loyalty	Out-going contact chain
**Breeding sites**				
Breeding and multiplier herds	intermediate	low	intermediate	high
Quarantine stations	intermediate	intermediate	intermediate	intermediate
Boar stations	intermediate	intermediate	high	low
**Production sites**				
Production herds	intermediate	low	high	intermediate
Weaner herds	high	low	intermediate	intermediate
Free-ranging pig herds	low	low	low	low
Organic pig herds	low	low	intermediate	low
**Hobby sites**				
Hobby herds	low	low	low	low
Pets	low	intermediate	high	low
Wild boar herds	low	low	low	low
Organic wild boar herds	not available	low	not available	low
**Transit sites**				
Traders	low	intermediate	low	low
Trade herds	low	intermediate	high	low
Pig shows	low	low	low	low
Livestock auctions	low	intermediate	low	high
Collection points (CP)	low	intermediate	intermediate	low
Slaughter animal markets	low	intermediate	low	low
**Miscellaneous**				
Zoos	low	low	low	low
Experimental facilities	low	low	low	low
**End of production sites**				
Slaughterhouses	low	high	high	low
Export isolation facilities	low	intermediate	high	low
CPs for dead animals	intermediate	low	intermediate	low
Cooling stations	high	low	high	low
Rendering plants	high	high	not available	low

Levels for in- and out-loyalty: low: mean < 0.45, intermediate: 0.45 ≤ mean ≤ 0.55, and high: mean > 0.55. Levels of the size of in-going and out-going contact chains: low: *m*_*ht*_ < 10, (2) intermediate: 10 ≤ *m*_*ht*_ ≤ 50, and high: *m*_*ht*_ > 50 with *m*_*ht*_ as mean value of the annual sizes of in-going and out-going contact chain per holding type.

**Fig 6:**
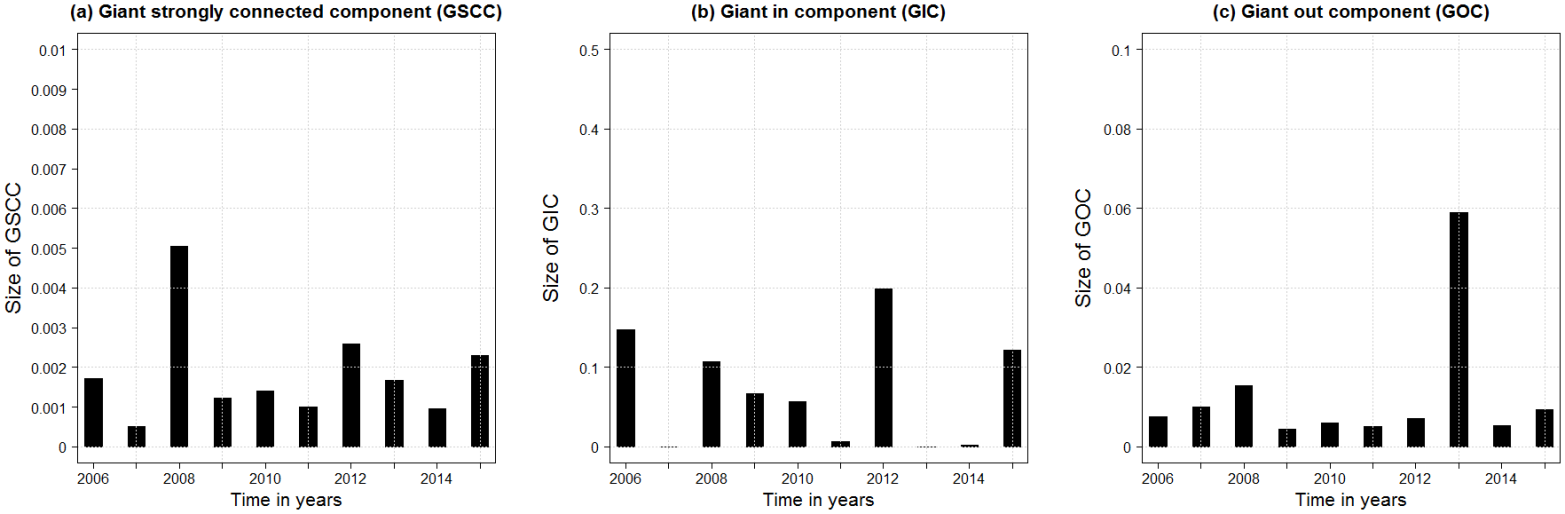
Pig movement network component sizes (proportion of overall number of active holdings). Sizes of (a) the giant connected components (GSCC), (b) the giant in components (GIC) and (c) the giant out components (GOC) for the yearly snapshots of the pig movement network in Denmark between 2006 and 2015.

The 10 years network showed a fragmentation index of 0.86. Thus the network is only connected to a low degree. Nevertheless, it contained (per definition) no isolated holdings. The fragmentation indices for the yearly network snapshots were 0.99 in all considered time periods. The sizes of the different GSCCs included less than 1% of active holdings each year (Fig 6), with the largest GSCC recorded in 2008 with 55 holdings. We observed variations between years in the size of the GIC, ranging from 1,893 holdings in 2006 to 2 holdings in 2007 (Fig 6). The size of the GOC was below 20% except in year 2013 (Fig 6).

Production herds constituted the highest proportion of holdings in the GSCC and were part of the GSCC in every year except of 2011 (Table 3). Breeding and multiplier herds were the only holding type that was part of the GIC in each year. Production herds, slaughterhouses and rendering plants were part of the GOC in every year, but also breeding and multiplier herds were part of GOC in 2011 and 2013.

**Table 3:** Number of years between 2006 and 2015, in which the holding type is present in the pig movement network components.

	Giant strongly connected component (GSCC)	Giant in component (GIC)	Giant out component (GOC)	Average number of active herds
**Breeding sites**				
Breeding and multiplier herds	3 (3)	10 (91)	2 (5)	264
Quarantine stations	1 (4)	7 (10)	4 (6)	52
Boar stations	1 (4)	4 (4)	3 (11)	17
**Production sites**				
Production herds	9 (15)	8 (708)	10 (74)	8343
Weaner herds	2 (1)	7 (34)	2 (6)	185
Free-ranging pig herds	4 (1)	6 (7)	4 (4)	191
Organic pig herds		3 (4)	4 (2)	93
**Hobby sites**				
Hobby herds	1 (1)	4 (3)	7 (5)	348
Pets			2 (1)	4
Wild boar herds		1 (1)		5
Organic wild boar herds				1
**Transit sites**				
Traders	2 (2)	4 (2)	5 (1)	7
Trade herds	2 (1)	2 (3)	2 (4)	12
Pig shows			2 (2)	7
Livestock auctions	1 (1)		1 (1)	1
Collection points (CP)	6 (2)	3 (1)	9 (5)	17
Slaughter animal markets			3 (2)	1
**Miscellaneous**				
Zoos			1	3
Experimental facilities				2
**End of production sites**				
Slaughterhouses			10 (24)	83
Export isolation facilities			1 (1)	3
CPs for dead animals				20
Cooling stations			1 (1)	13
Rendering plants			10 (1)	1
**Average component size**	18	529	120	

Number of years of the considered time period of 10 years from 2006 to 2015 in which a certain holding type was part of the giant strongly connected component (GSCC), the giant in component (GIC) and the giant out component (GOC). Values in brackets show the average number of holdings present in the component. Additionally, the average number of active herds is shown.

#### Temporal network analysis

**Fig 7:**
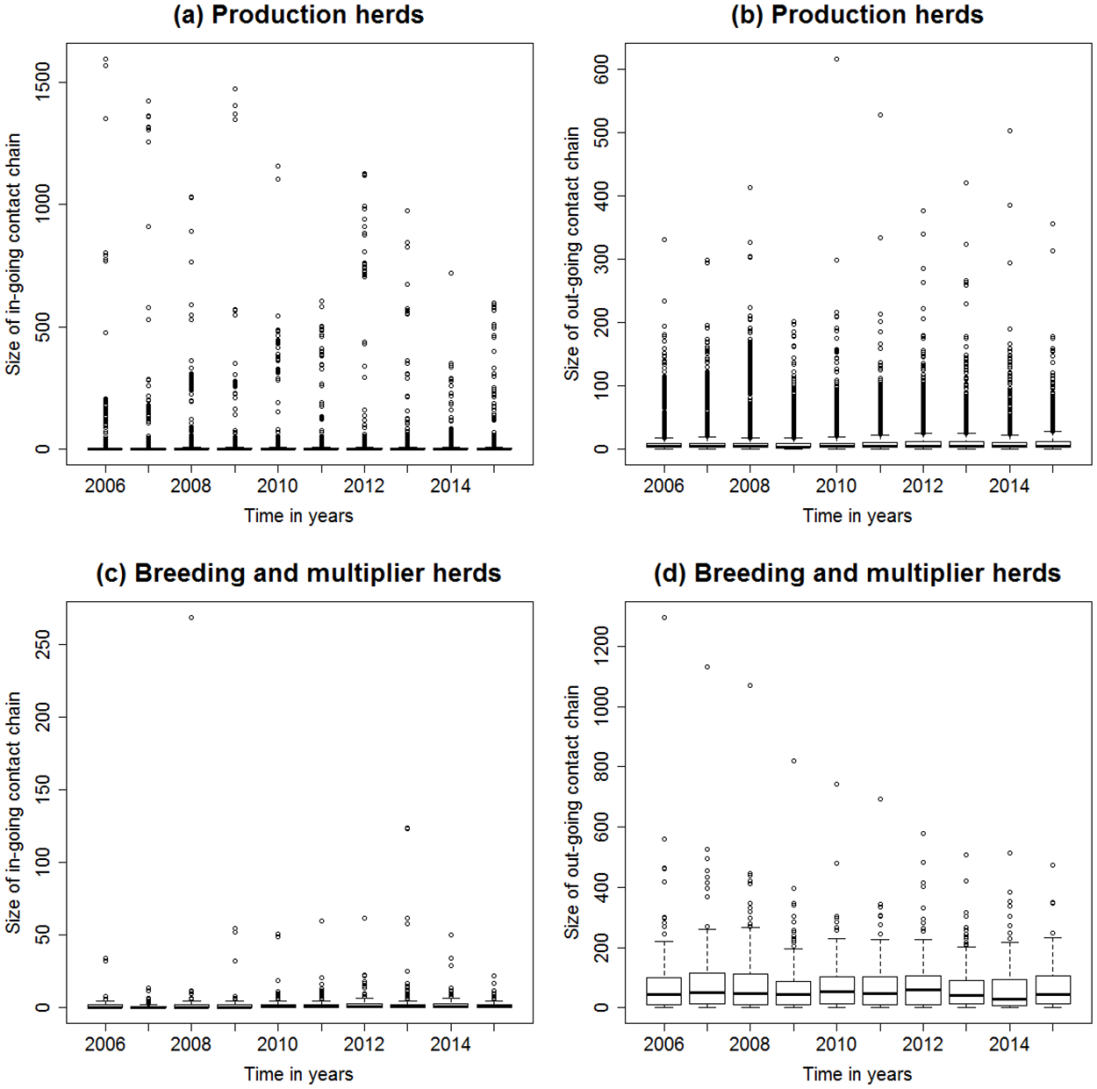
Contact chains for production and breeding and multiplier herds. Boxplots of (a) in-going and (b) out-going contact chains for production herds and (c) in-going and (d) out-going contact chains for breeding and multiplier herds from 2006 to 2015.

We calculated the size of the in-going and out-going contact chains for the whole network from 2006 to 2015 (S4 File). In total, the values of the in-going contact chains were higher compared to the size of the out-going contact chains over the study period of 10 years. The size of the in-going and out-going contact chains varied between holding types (Fig 7, Table 2, and S4 File). All production sites showed low levels of in-going contact chains, whereas the levels of out-going contact chains vary. Quarantine and boar stations showed intermediate levels of in-going contact chains and we observed a high level of out-going contact chain for breeding and multiplier herds. Except of livestock auctions, all transit sites also show low levels of out-going contact chains.

## Discussion

### Data set

Although there have been many studies analysing pig movement networks, most covered only time periods of one to three years [12,25,26,27]. The presented study describes the development of the number of active pig holdings, holding sizes and the network of pig movements in Denmark over a period of 10 years from 1^st^ January 2006 to 31^st^ December 2015, and hence presents long term changes.

The pig movements between holdings included in this study comprised over 99% of the movement records in the database. Only holdings not registered as pig holdings in the CHR and registration errors in the movement records were excluded. The registration errors mainly were related to out-going movements of slaughterhouses. As slaughterhouses are dead ends of the production chain [28] they do not play a key role in disease spreading. Exclusion of these records is therefore not expected to influence the overall results of this study.

The holding types are self-reported by the owner of the holding. As there are no definitions of holding types available, misclassification is likely. Additionally, available holding types in the CHR changed over time. The holding type “livestock auctions” does not exist anymore since 2008, as pigs are no longer sold at auctions. The holding type “organic wild boar” was introduced in 2015. Breeding and multiplier herds are summarized in one category. Breeding herds are not expected to have in-going contacts and differ from multiplier herds regarding herd management. This fact becomes important when developing control programs for different holding types. Furthermore, the validity of the CHR has increased over the time period, as the CHR data has been merged with other types of data with the purpose of decreasing the use of antimicrobials [29], which might influence the number of herds in each holding type.

It might be useful to add clear definitions for the holding types to the CHR and restrict the use of some holding types dependent on other available information such as the registration of the holding as specific pathogen free herd (SPF). Such definitions might also include information on the biosecurity level of the holding. Information on biosecurity in Danish pig holdings are currently only available for SPF herds, and registered in the private SPF-register. Furthermore, a non-SPF status cannot be interpreted as low level of biosecurity, as many holdings have high biosecurity standards, even if not enrolled in the SPF system. However, implementation of biosecurity in the CHR register could add important information regarding risk of disease introduction and spread. However, the effect of biosecurity depends on a daily continual awareness of biosecurity procedures. Self-reporting of the level of biosecurity might lead to overestimation of one’s own performance, like the lack of control might lead to a slowly decrease in the producer’s awareness and performance regarding biosecurity. Official veterinarians could perform regular inspections and use already available methods to assign a biosecurity level to each holding.

### Data analysis

#### Data summaries

We estimated the number of weaners per holding in 2006, because these values were not given in the data. The estimated holding sizes fit into the observed trend, even though higher values have been reported from other countries for estimates of the number of weaners based on the number of sows per holding [30]. Nevertheless, these values might have changed since 2006.

We observed a decreasing number of holdings, but an increasing size of the holdings in the considered time period. Nöremark et al. [9] observed the same trend in Sweden. The decreasing frequency of movements is most likely due to the decreasing number of active holdings during the study period. Lentz et al. [12] observed similar patterns for the frequency of movements for a German pig movement network. In addition, the median size of batches of pigs moved remained constant.

The maximum batch sizes were very high in each year and this could be attributed to errors in reporting: movements could have occurred over a certain time period but were all reported on a single day. Nevertheless, larger batches might increase the probability of transferring a disease from one holding to another.

The constant average number of out-movements from breeding and multiplier herds and production herds in combination with the reduction of movements to slaughterhouses and rendering plants could be explained by the increased export. Furthermore, the reduction of movements to transit sites might influence the risk of introduction and spread of diseases. The increase in movements from weaner herds might describe a trend towards a more specialized production with more locations.

The type of holding highly influenced the frequency of contacts with other holdings as well as to which type of holding contacts occurred, which reflects the pyramidal structure of the Danish pig production sector. Lindström et al. [26] highlighted with a simulation study that these contact patterns might result in substantial differences in disease transmission via animal movements, depending on the index holding.

#### Network analysis

Both, static and temporal network analysis approaches were used to describe the Danish pig movement data, as the static view alone does not take the dynamic aspects of contact patterns into account [31].

There are several methods available in both, static and temporal network analysis. We investigated in- and out-loyalty and in-going and out-going contact chains for 24 holding types. Other static network approaches such as in- and out-degree were shown to be less informative to describe the potential risk of a holding in spreading or contracting a disease [32]. Indirect in- and out-going contacts should be taken into account to better evaluate the risk of each holding becoming infected or spreading infections [33]. Thus, in this study we calculated the in-going and out-going contact chain taking the temporal order of pig movements into account. Additionally, [31,33] describe the out-going infection chain as more reasonable estimate of a potential maximal epidemic size. Nevertheless, we used a time frame of one year to calculate the contact chains. If a certain disease is considered, the time frame needs to be adapted to a meaningful range reflecting the duration of the incubation and infectious periods of that disease.

The methodological framework for the analysis of temporal networks is still in a starting phase [34]. The concept of components is well understood in static networks. Even though it is still a challenge to transfer the idea to temporal networks, an understanding of the static component structure is useful to develop meaningful surveillance and control programs. The small sizes of the yearly GSCCs might result from the pyramid structure or so called tree-like structure of the Danish pig production sector and lead to a restriction of the size of disease outbreaks [12]. Nevertheless, these estimates might be overestimated because time is not considered. Most animal movement networks are highly fragmented, even if longer time periods are considered [33].

Even though we observed variability of loyalty values between holdings of the same type, clear loyalty patterns for different holding types were found. All breeding sites showed an intermediate level of in-loyalty whereas there was more variation on the level of in-loyalty for production sites. This reflects different management structures of holding types, which should be taken into account when developing control programs to limit the spread of diseases. Hobby and transit sites show lower levels of in-loyalty and thus might be at higher risk for introduction of diseases. On the other hand, only trade herds show a high out-loyalty as the majority of out-going movements go either to slaughterhouses or rendering plants. All other transit sites showed lower levels of out-loyalty which could increase the potential of spreading pathogens via trade.

Also the sizes of in- and out-going contact chain vary between holding types. End of production sites (Fig 1) show the highest levels of in-going contact chains, whereas we observed low levels for production sites. Breeding sites had higher out-going contact chains compared to all other holding type categories. Both reflect the pyramidal structure of the underlying network. Büttner et al. [32] investigated the size of in-going and out-going contact chain for four pig producing holding types in northern Germany (multipliers, farrowing farms, finisher farms and farrow-to-finisher farms). The values are lower compared to the values observed in this present study. This might be due to the lower number of active holdings within the study area and the considered time period of 3 years in [32].

### Implications for disease control

Bigras-Poulin et al. [16] described the Danish pig movement network as one where a pathogen can maintain itself and spread even at low prevalences and thus eradication of pathogens would be difficult. Our results support these findings as we found holdings with high values of out-going contact chains that might have a high potential of spreading pathogens in the pig movement network. Additionally, the yearly components indicate circular connections between holdings that might support pathogen perpetuation. Nevertheless, the true transmission probability of infectious diseases via pig movements depends also on disease and holding specific parameters (e.g. virulence, biosecurity level and farmers’ behaviour). Moreover, one should take into account the holding types present in the GIC and GSCC, as (1) these holdings might reach more other holdings, (2) the potential of an introduction of a pathogen to the holdings in a GSCC is related to the size of the GIC and (3) the maintenance of a pathogen is related to the size of the GSCC.

Holdings with a combination of a low/intermediate out-loyalty and high level of out-going contact chains might have a higher risk of spreading diseases compared to holdings with a high out-loyalty and a low size of out-going contact chains and should thus be targeted by surveillance. In our study breeding and multiplier herds at the top of the production pyramid showed this combination of high risk potential for disease spreading. Danish breeding and multiplier herds have very high levels of biosecurity, and therefore a somewhat lower risk of introduction of pathogens. However, once the pathogen is introduced, the following risk of spread is high. In the SPF system, these herds have monthly veterinary visits with focus on herd health and biosecurity [35]. Holdings registered as quarantine stations and weaner herds showed an intermediate risk, which may have the potential for disease spread and maintenance. Naturally, quarantine stations are under special surveillance as to assure the animals are free from disease before entrance to boar stations or other holdings [36,37].

In-loyalty and the size of the in-going contact chain are linked to the risk of the introduction of diseases via pig movements. Thus, high/intermediate in-loyalty combined with a low level of in-going contact chains might present a smaller risk compared to low in-loyalty and a high level of in-going contact chains. Breeding and multiplier herds, production and weaner herds showed the described lower risk combination. Production herds showed a low risk of receiving and spreading of pathogens, nevertheless, they constituted the largest group in the GSCC and GOC. As holdings in these components are linked to a wide range of other holdings and holding types, the risk for spreading and receiving diseases via pig movements might be still remarkable.

As the Danish pig production system is organised in a pyramidal structure with breeding herds at the top and slaughter pig production at the bottom [16], the start of eradication and control could be started in breeding herds. Restricted trade between holdings tested positive to holdings tested negative for a considered disease, as in the SPF-system, might additionally reduce the risk of introduction and re-introduction. This approach was recommended for LA-MRSA in [38] and could also be applicable for other endemic diseases. Nevertheless, for each considered disease, the analysis needs to be adapted regarding the time period for which loyalty and contact chains should be calculated. Based on this temporal adaptation, new definitions for the categorization of holding types should be used.

Around one third of the connections between holdings persisted between 2006 and 2015, suggesting long lasting trade connections are in place. This may lead to compliance issues if the disease status of holdings needs to be considered before movements without legislation in place to restrict these movements. This could be especially true for endemic diseases: intensive information and financial compensation might help to increase the motivation to change to a supplier that tested negative to prevent disease spread from positive to negative herds. Nevertheless, investigating loyalty patterns of holdings might help to find trade connections of positive tested herds and thus limiting the spread of pathogens in the network.

As animal trade is not the only pathway for transmission of pathogens [1,39], focussing only on holdings showing a high potential of spreading a pathogen might not be successful. Thus if eradication of a certain disease is the purpose, potential infection via mechanisms other than animal movements should be considered. Simulation modelling of the spread of pathogens could be based on animal movements and take into account other transmission mechanisms. The aim of a simulation model could be to understand the spread of a pathogen, but also to develop and test meaningful surveillance and control programs based on simulation studies. A first and main step in order to develop a simulation model mimicking the spread of pathogens is to describe animal movements and understand the movement patterns.

## Supporting information

S1 File Holding sizes of active Danish pig holdings. The file includes supporting figures and tables related to the holding sizes:
Descriptive statistics of the trends (1) of the holding sizes of active Danish pig holdings (holdings that at least once send or receive pigs to or from another holding) between 1^st^ January 2006 and 31^st^ December 2015 in Denmark (Table 1),Proportion of holdings active between 1^st^ January 2006 and 31^st^ December 2015 in Denmark, categorized by the size of holding (Table 2),Holding sizes by holding types for (a) breeding sites, (b) production sites, (c) hobby sites, (d) transit sites, (e) miscellaneous sites, and (f) end of production sites (Fig 1),Descriptive statistics of the trend of all 24 holding types (Tables 3–8).
(PDF)

S2 File Batch sizes and number of pig movements per holding type. The file includes supporting tables related to the number of pigs moved per registered pig movement (batch size) and the number of pig movements per holding type:
Descriptive statistics of the development of the number of pigs per movement (batch size) of registered pig movements between 1^st^ January 2006 and 31^st^ December 2015 in Denmark (Table 1).Median batch size by holding type of sending and receiving holding of registered pig movements between 1^st^ January 2006 and 31^st^ December 2015 in Denmark (Tables 2 and 3).Average number of pig movements per holding type of sending and receiving holding per year between 1^st^ January 2006 and 31^st^ December 2015 in Denmark (Tables 4 and 5).
(PDF)

S3 File In- and out-loyalty per holding type. The file includes supporting figures and tables related to the in- and out-loyalty of the investigated holding types:
In-loyalty for each pair of consecutive years for the whole network of pig movements from 1^st^ January 2006 to 31^st^ December 2015 in Denmark (Figure 1),Out-loyalty for each pair of consecutive years for the whole network of pig movements from 1^st^ January 2006 to 31^st^ December 2015 in Denmark (Figure 2).In- and out-loyalty for (a) breeding sites, (b) production sites, (c) hobby sites, (d) transit sites, (e) miscellaneous sites, and (f) end of production sites (Figures 3–8),Descriptive summaries of in- and out-loyalty per holding type (Tables 1 and 2).
(PDF)

S4 File Contact chains. The file includes supporting figures and tables related to the in-going and out-going contact chains:
Size of (a) in-going and (b) out-going contact chain for the whole pig movement network in Denmark from 1^st^ January 2006 to 31^st^ December 2015 (Figure 1).In-going and out-going contact chains for (a) breeding sites, (b) production sites, (c) hobby sites, (d) transit sites, (e) miscellaneous sites, and (f) end of production sites (Figures 2–7).
(PDF)
