# Supplementary material for: Network analysis of pig movements: Loyalty patterns and contact chains of different holding types in Denmark
Source: PLoS One. 2017 Jun 29;12(6):e0179915. doi: 10.1371/journal.pone.0179915 (PMC5491064; doi:10.1371/journal.pone.0179915)
Supplement: S1 File — The file includes supporting figures and tables related to the holding sizes: Descriptive statistics of the trends (1) of the holding sizes of active Danish pig holdings (holdings that at least once send or receive pigs to or from another holding) between 1st January 2006 and 31st December 2015 in Denmark (Table 1),Proportion of holdings active between 1st January 2006 and 31st December 2015 in Denmark, categorized by the size of holding (Table 2),Holding sizes by holding types for (a) breeding sites, (b) production sites, (c) hobby sites, (d) transit sites, (e) miscellaneous sites, and (f) end of production sites (Fig 1),Descriptive statistics of the trend of all 24 holding types (Tables 3–8). (PDF) [file pone.0179915.s001.pdf]

S1 File. Size of holdings.

Table 1. Size of holdings of active Danish pig holdings. Descriptive statistics of the holding sizes of active Danish pig holdings (holdings that at least once send or receive pigs to or from another holding) between 1 Jan 2006 and 31 Dec 2015 in Denmark. If no sows, finishers and weaners were registered, the total holding size was set to “not available”.

	Number of active holdings	Minimum	1st Quantile	Median	Mean	3rd Quantile	Maximum	Not available
**2006**	12,814	1	252	816	1,227	1,737	15,750	880
**2007**	11,920	1	300	823	1,254	1,700	29,940	398
**2008**	10,886	1	395	1,000	1,362	1,900	29,940	390
**2009**	9,816	1	440	1,086	1,474	2,000	29,940	392
**2010**	9,270	1	500	1,200	1,566	2,121	29,940	374
**2011**	8,875	1	520	1,200	1,598	2,230	23,950	342
**2012**	8,502	1	600	1,300	1,668	2,300	21,000	561
**2013**	8,370	1	650	1,400	1,742	2,400	21,000	952
**2014**	8,366	1	700	1,500	1,796	2,430	21,000	1,296
**2015**	7,835	1	600	1,410	1,813	2,500	19,620	536

Table 2. Proportion of active holdings by holding size. Proportion of holdings active between 1 Jan 2006 and 31 Dec 2015 in Denmark, categorized by the size of holding.

Herdsize	2006	2007	2008	2009	2010	2011	2012	2013	2014	2015
**0**	6.87	3.34	3.58	3.99	4.03	3.85	6.60	11.37	15.49	6.84
**0 - 1,000**	53.71	56.08	50.35	46.38	43.39	41.72	38.44	33.91	30.67	36.23
**1,001 - 2,000**	21.62	23.00	25.03	25.96	26.67	26.63	26.28	25.89	25.26	25.45
**2,001 - 3,000**	9.42	10.19	12.32	13.47	14.36	15.13	15.28	15.33	15.14	15.33
**3,001 - 4,000**	4.39	3.47	4.77	5.45	6.08	6.58	7.00	7.00	6.97	7.76
**4,001- 5,000**	1.76	1.61	1.78	2.25	2.47	2.96	3.16	3.23	3.18	3.87
**5,001 - 6,000**	0.99	0.74	0.92	1.10	1.39	1.58	1.53	1.51	1.53	1.83
**6,001 - 7,000**	0.66	0.36	0.42	0.47	0.59	0.70	0.69	0.73	0.72	1.03
**7,001 - 8,000**	0.23	0.26	0.23	0.25	0.29	0.34	0.47	0.48	0.48	0.65
**8,001 - 9,000**	0.09	0.18	0.13	0.14	0.17	0.12	0.15	0.16	0.16	0.32
**9,001 - 10,000**	0.07	0.12	0.06	0.10	0.12	0.07	0.09	0.10	0.10	0.27
**10,001 - 11,000**	0.07	0.09	0.10	0.08	0.09	0.11	0.12	0.12	0.12	0.14
**11,001 - 12,000**	0.05	0.11	0.05	0.04	0.05	0.08	0.09	0.10	0.10	0.13
**12,001 - 13,000**	0.03	0.10	0.05	0.05	0.05	0.01	0.02	0.02	0.02	0.08
**13,001 - 14,000**		0.06	0.03	0.02	0.02	0.05	0.04	0.04	0.04	0.01
**14,001 - 15,000**	0.02	0.08	0.02	0.03	0.04	0.01	0.01	0.01	0.01	0.03
**15,001 - 16,000**	0.02	0.02				0.01				
**16,001 - 17,000**		0.06	0.04	0.03	0.01	0.01	0.01	0.01	0.01	
**17,001 - 18,000**		0.04	0.01	0.02	0.01					0.01
**18,001 - 19,000**		0.03	0.03	0.02						
**19,001 - 20,000**		0.01	0.03	0.03	0.03	0.01				0.03
**20,001 - 21,000**		0.01	0.02	0.02	0.02	0.01	0.01	0.01	0.01	
**21,001 - 22,000**										
**22,001 - 23,000**			0.01							
**23,001 - 24,000**			0.02	0.02	0.02	0.01				
**24,001 - 25,000**		0.01	0.01	0.03	0.04					
**25,001 - 26,000**		0.01								
**26,001 - 27,000**		0.01	0.01	0.01	0.01					
**27,001 - 28,000**										
**28,001 - 29,000**				0.01	0.01					
**29,001 - 30,000**		0.01	0.01	0.01	0.01					

Figure 1. Holding sizes by holding type. Median (solid line) and average (dashed line) of holding sizes for (a) breeding sites, (b) production sites, (c) hobby sites, (d) transit sites, and (e) miscellaneous sites. Coloured areas represent the range between 1st and 3rd quantile. Values for holding types not shown in this figure are not representative and shown in Table 3-8.

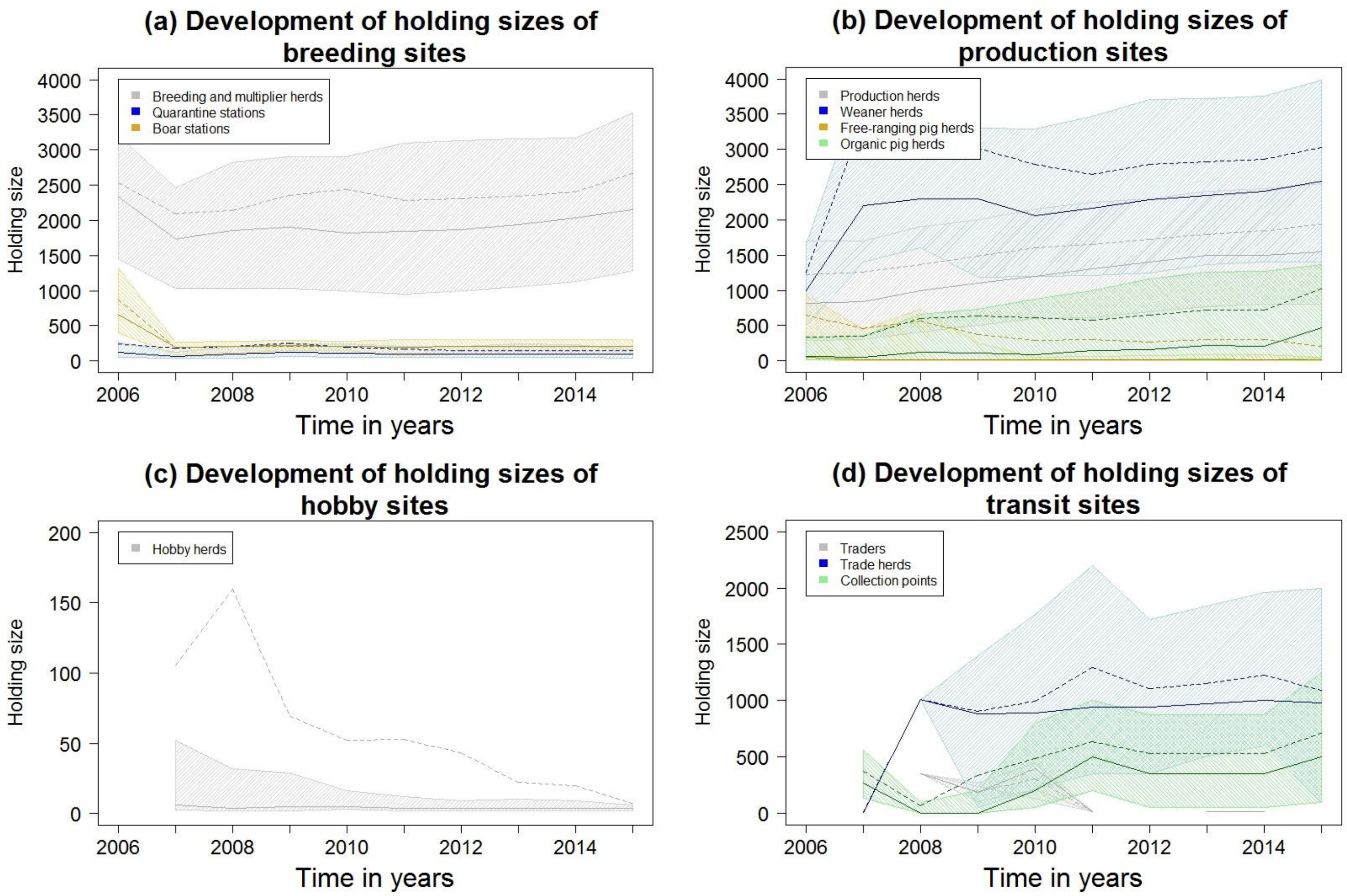

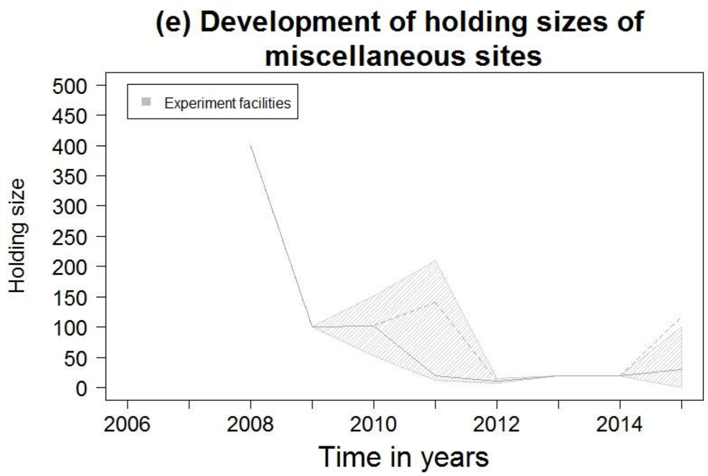
(e) Development of holding sizes of miscellaneous sites
Experiment facilities
Holding size
500
450
400
350
300
250
200
150
100
50
0
2006
2008
2010
2012
2014
Time in years

Table 3. Descriptive summaries of holding sizes for breeding sites.

		Number of active holdings	Minimum	1st quantile	Median	Mean	3rd quantile	Maximum	Not available
Breeding and multiplier herds	2006	278	6	1,450	2,330	2,532	3,204	12,580	18
	2007	261	2	1,028	1,740	2,097	2,465	18,500	2
	2008	252	2	1,028	1,860	2,141	2,828	10,950	1
	2009	263	2	1,026	1,900	2,354	2,910	24,890	2
	2010	270	2	1,000	1,825	2,442	2,910	24,890	1
	2011	276	2	950	1,846	2,291	3,102	19,200	0
	2012	272	1	1,000	1,870	2,308	3,138	10,050	4
	2013	271	1	1,050	1,940	2,346	3,153	10,050	12
	2014	260	1	1,132	2,040	2,404	3,170	10,050	16
	2015	234	1	1,280	2,150	2,663	3,530	11,950	1
Quarantine stations	2006	71	1	50	125	240	267	2,005	11
	2007	75	1	40	60	178	125	2,960	4
	2008	62	1	33	100	209	205	3,200	2
	2009	57	3	60	125	250	240	3,200	6
	2010	48	3	50	105	192	243	1,250	4
	2011	39	3	45	98	172	205	2,000	3
	2012	46	10	48	100	145	200	700	5
	2013	41	10	45	95	147	240	700	4
	2014	36	1	47	100	146	230	700	1
	2015	44	3	40	95	150	160	600	3
Boar stations	2006	18	52	396	660	881	1,320	2,046	3
	2007	17	20	85	197	189	271	393	0
	2008	16	26	108	211	207	278	393	0
	2009	15	26	145	215	210	285	393	0
	2010	16	26	140	206	206	278	393	0
	2011	16	25	72	200	194	299	405	0
	2012	17	26	95	202	200	298	405	0
	2013	17	26	95	202	200	298	405	0
	2014	17	26	95	202	200	298	405	0
	2015	17	26	95	202	203	298	405	0

Table 4. Descriptive summaries of holding sizes for production sites.

		Number of active holdings	Minimum	1st quantile	Median	Mean	3rd quantile	Maximum	Not available
Production herds	2006	11733	1	287	817	1,222	1,700	15,750	548
	2007	10867	1	300	845	1,253	1,700	29,940	193
	2008	9902	1	410	1,000	1,361	1,900	29,940	195
	2009	8691	1	500	1,100	1,482	2,000	29,940	196
	2010	7940	1	600	1,200	1,601	2,150	29,940	161
	2011	7538	1	614	1,300	1,647	2,250	23,950	110
	2012	7090	1	700	1,400	1,723	2,303	21,000	200
	2013	6819	1	767	1,500	1,792	2,400	21,000	355
	2014	6623	1	800	1,500	1,844	2,440	21,000	444
	2015	6230	1	800	1,550	1,938	2,510	19,620	110
Weaner herds	2006	110	30	500	1,000	1,241	1,650	5,080	47
	2007	105	30	1,400	2,200	3,431	4,000	26,600	0
	2008	106	1	1,600	2,300	3,377	3,625	26,600	2
	2009	171	98	1,188	2,300	3,018	3,300	26,600	0
	2010	241	12	1,200	2,062	2,789	3,285	26,600	1
	2011	240	1	1,210	2,162	2,648	3,475	12,400	2
	2012	227	3	1,249	2,288	2,782	3,706	12,400	3
	2013	224	3	1,362	2,345	2,821	3,726	12,400	6
	2014	223	3	1,407	2,400	2,860	3,751	12,400	12
	2015	201	7	1,400	2,545	3,028	3,989	13,000	2
Free-ranging pig herds	2006	325	1	10	52	644	936	8,000	45
	2007	279	1	4	20	458	450	10,330	26
	2008	189	1	5	18	559	729	8,255	22
	2009	190	1	5	18	378	250	5,495	25
	2010	157	1	4	11	291	54	5,495	19
	2011	145	1	4	12	304	60	5,495	19
	2012	141	1	4	14	262	72	3,500	35
	2013	153	1	4	14	300	83	3,500	66
	2014	169	1	4	13	300	83	3,500	82
	2015	164	1	3	10	204	55	4,350	36

Organic herds	2006	76	1	14	65	338	331	3,080	9
	2007	79	1	10	45	343	350	3,835	6
	2008	117	1	10	118	595	658	10,070	5
	2009	106	1	15	115	640	738	6,297	4
	2010	99	1	12	83	610	875	6,297	6
	2011	93	2	12	140	573	1,000	3,150	4
	2012	90	1	13	159	649	1,162	5,495	6
	2013	87	1	22	223	722	1,261	5,495	11
	2014	90	3	20	210	717	1,272	5,495	15
	2015	88	1	41	470	1,021	1,360	8,490	5

Table 5. Descriptive summaries of holding sizes for hobby sites.

		Number of active holdings	Minimum	1st quantile	Median	Mean	3rd quantile	Maximum	Not available
Hobby herds	2006	69	NA	NA	NA	NA	NA	NA	69
	2007	101	1	3	6	105	52	1,600	45
	2008	110	1	2	4	160	32	3,600	43
	2009	196	1	2	5	69	29	2,150	55
	2010	358	1	3	5	52	16	1,585	79
	2011	391	1	2	4	53	12	1,810	104
	2012	454	1	2	4	43	9	3,027	178
	2013	561	1	2	4	22	10	830	335
	2014	721	1	2	4	20	9	830	531
	2015	521	1	2	4	7	6	281	96
Pets	2006	5	NA	NA	NA	NA	NA	NA	5
	2007	2	NA	NA	NA	NA	NA	NA	2
	2008	1	NA	NA	NA	NA	NA	NA	1
	2009	1	NA	NA	NA	NA	NA	NA	1
	2010	2	500	688	875	875	1,062	1,250	0
	2011	2	350	350	350	350	350	350	1
	2012	1	NA	NA	NA	NA	NA	NA	1
	2013	3	NA	NA	NA	NA	NA	NA	3
	2014	10	NA	NA	NA	NA	NA	NA	10
	2015	11	1	1	1	4	3	13	6
Wild boar herds	2006	0	NA	NA	NA	NA	NA	NA	0
	2007	2	NA	NA	NA	NA	NA	NA	2
	2008	1	NA	NA	NA	NA	NA	NA	1
	2009	1	NA	NA	NA	NA	NA	NA	1
	2010	1	NA	NA	NA	NA	NA	NA	1
	2011	1	NA	NA	NA	NA	NA	NA	1
	2012	15	4	4	4	4	4	4	14
	2013	8	NA	NA	NA	NA	NA	NA	8
	2014	8	NA	NA	NA	NA	NA	NA	8
	2015	11	2	4	13	25	48	72	1

Organic wild boar herds	2006	0	NA	NA	NA	NA	NA	NA	0
	2007	0	NA	NA	NA	NA	NA	NA	0
	2008	0	NA	NA	NA	NA	NA	NA	0
	2009	0	NA	NA	NA	NA	NA	NA	0
	2010	0	NA	NA	NA	NA	NA	NA	0
	2011	0	NA	NA	NA	NA	NA	NA	0
	2012	0	NA	NA	NA	NA	NA	NA	0
	2013	0	NA	NA	NA	NA	NA	NA	0
	2014	0	NA	NA	NA	NA	NA	NA	0
	2015	1	8	8	8	8	8	8	0

Table 6. Descriptive summaries of holding sizes for transit sites.

		Number of active holdings	Minimum	1st quantile	Median	Mean	3rd quantile	Maximum	Not available
Traders	2006	7	NA	NA	NA	NA	NA	NA	7
	2007	6	NA	NA	NA	NA	NA	NA	6
	2008	10	350	350	350	350	350	350	9
	2009	8	20	103	185	185	268	350	6
	2010	8	20	210	400	307	450	500	5
	2011	5	20	20	20	20	20	20	4
	2012	6	NA	NA	NA	NA	NA	NA	6
	2013	7	20	20	20	20	20	20	6
	2014	8	20	20	20	20	20	20	7
	2015	3	NA	NA	NA	NA	NA	NA	3
Trade herds	2006	0	NA	NA	NA	NA	NA	NA	0
	2007	1	10	10	10	10	10	10	0
	2008	1	1,010	1,010	1,010	1,010	1,010	1,010	0
	2009	7	14	58	880	909	1,400	2,555	0
	2010	20	2	219	890	998	1,765	2,555	0
	2011	17	1	350	940	1,294	2,200	4,501	0
	2012	17	2	350	940	1,107	1,720	2,900	0
	2013	16	2	513	971	1,154	1,840	2,900	0
	2014	15	2	600	1,002	1,230	1,960	2,900	0
	2015	16	2	100	983	1,090	2,000	2,900	2
Pig shows	2006	9	1	1	1	1	1	1	8
	2007	8	1	6	10	7	11	11	5
	2008	8	1	1	1	1	1	1	6
	2009	6	1	4	6	6	9	11	4
	2010	6	1	4	6	6	9	11	4
	2011	6	1	4	6	6	9	11	4
	2012	9	1	4	6	6	9	11	7
	2013	9	1	4	6	6	9	11	7
	2014	6	1	4	6	6	9	11	4
	2015	7	1	4	6	6	9	11	5

Livestock auctions	2006	1	NA	NA	NA	NA	NA	NA	1
	2007	1	NA	NA	NA	NA	NA	NA	1
	2008	0	NA	NA	NA	NA	NA	NA	0
	2009	0	NA	NA	NA	NA	NA	NA	0
	2010	0	NA	NA	NA	NA	NA	NA	0
	2011	0	NA	NA	NA	NA	NA	NA	0
	2012	0	NA	NA	NA	NA	NA	NA	0
	2013	0	NA	NA	NA	NA	NA	NA	0
	2014	0	NA	NA	NA	NA	NA	NA	0
	2015	0	NA	NA	NA	NA	NA	NA	0
Collection points (CP)	2006	7	NA	NA	NA	NA	NA	NA	7
	2007	11	1	136	270	374	560	850	8
	2008	15	1	1	1	67	101	200	12
	2009	16	1	1	3	341	200	1,500	11
	2010	17	1	51	200	484	800	1,500	11
	2011	17	1	200	500	640	1,000	1,500	12
	2012	20	1	51	350	534	875	1,500	14
	2013	21	1	51	350	534	875	1,500	15
	2014	24	1	51	350	534	875	1,500	18
	2015	25	1	101	500	715	1,250	1,800	18
Slaughter animal markets	2006	1	NA	NA	NA	NA	NA	NA	1
	2007	2	NA	NA	NA	NA	NA	NA	2
	2008	2	NA	NA	NA	NA	NA	NA	2
	2009	0	NA	NA	NA	NA	NA	NA	0
	2010	0	NA	NA	NA	NA	NA	NA	0
	2011	0	NA	NA	NA	NA	NA	NA	0
	2012	0	NA	NA	NA	NA	NA	NA	0
	2013	1	NA	NA	NA	NA	NA	NA	1
	2014	1	NA	NA	NA	NA	NA	NA	1
	2015	1	NA	NA	NA	NA	NA	NA	1

Table 7. Descriptive summaries of holding sizes for miscellaneous sites.

		Number of active holdings	Minimum	1st quantile	Median	Mean	3rd quantile	Maximum	Not available
Zoos	2006	0	NA	NA	NA	NA	NA	NA	0
	2007	0	NA	NA	NA	NA	NA	NA	0
	2008	0	NA	NA	NA	NA	NA	NA	0
	2009	0	NA	NA	NA	NA	NA	NA	0
	2010	0	NA	NA	NA	NA	NA	NA	0
	2011	1	20	20	20	20	20	20	0
	2012	1	NA	NA	NA	NA	NA	NA	1
	2013	4	20	20	20	20	20	20	3
	2014	4	NA	NA	NA	NA	NA	NA	4
	2015	4	3	4	5	5	6	6	0
Experimental facilities	2006	0	NA	NA	NA	NA	NA	NA	0
	2007	0	NA	NA	NA	NA	NA	NA	0
	2008	1	400	400	400	400	400	400	0
	2009	1	100	100	100	100	100	100	0
	2010	3	3	52	102	102	151	200	1
	2011	3	4	12	20	141	210	400	0
	2012	2	3	7	12	12	16	20	0
	2013	1	20	20	20	20	20	20	0
	2014	2	20	20	20	20	20	20	1
	2015	5	1	1	30	116	100	450	0

Table 8. Descriptive summaries of holding sizes for end of production sites.

		Number of active holdings	Minimum	1st quantile	Median	Mean	3rd quantile	Maximum	Not available
Slaughterhouses	2006	100	1	3	4	38	57	110	97
	2007	97	1	4	1,800	1,854	1,975	5,491	92
	2008	89	1	45	870	885	1,710	1,800	85
	2009	83	1	1	1	561	841	1,680	80
	2010	80	1	1	1	1	1	1	78
	2011	78	1	1	1	1	1	1	76
	2012	74	1	1	1	1	1	1	72
	2013	75	1	1	1	1	1	1	73
	2014	77	1	1	1	1	1	1	75
	2015	76	1	1	1	1	1	1	75
Export isolation facilities	2006	0	NA	NA	NA	NA	NA	NA	0
	2007	0	NA	NA	NA	NA	NA	NA	0
	2008	0	NA	NA	NA	NA	NA	NA	0
	2009	0	NA	NA	NA	NA	NA	NA	0
	2010	0	NA	NA	NA	NA	NA	NA	0
	2011	3	150	165	180	260	315	450	0
	2012	3	27	104	180	219	315	450	0
	2013	3	27	104	180	219	315	450	0
	2014	3	27	104	180	219	315	450	0
	2015	3	27	104	180	219	315	450	0
CPs for dead animals	2006	2	NA	NA	NA	NA	NA	NA	2
	2007	4	6	11	16	16	20	25	2
	2008	2	NA	NA	NA	NA	NA	NA	2
	2009	2	240	280	320	320	360	400	0
	2010	2	25	25	25	25	25	25	1
	2011	2	1,620	1,620	1,620	1,620	1,620	1,620	1
	2012	7	10	83	155	155	228	300	5
	2013	18	10	83	155	155	228	300	16
	2014	35	10	83	155	155	228	300	33
	2015	121	300	300	300	300	300	300	120

Cooling stations	2006	1	NA	NA	NA	NA	NA	NA	1
	2007	1	NA	NA	NA	NA	NA	NA	1
	2008	1	NA	NA	NA	NA	NA	NA	1
	2009	1	180	180	180	180	180	180	0
	2010	1	NA	NA	NA	NA	NA	NA	1
	2011	1	420	420	420	420	420	420	0
	2012	9	NA	NA	NA	NA	NA	NA	9
	2013	30	NA	NA	NA	NA	NA	NA	30
	2014	33	NA	NA	NA	NA	NA	NA	33
	2015	51	NA	NA	NA	NA	NA	NA	51
Rendering plants	2006	1	NA	NA	NA	NA	NA	NA	1
	2007	1	NA	NA	NA	NA	NA	NA	1
	2008	1	NA	NA	NA	NA	NA	NA	1
	2009	1	NA	NA	NA	NA	NA	NA	1
	2010	1	NA	NA	NA	NA	NA	NA	1
	2011	1	NA	NA	NA	NA	NA	NA	1
	2012	1	NA	NA	NA	NA	NA	NA	1
	2013	1	NA	NA	NA	NA	NA	NA	1
	2014	1	NA	NA	NA	NA	NA	NA	1
	2015	1	NA	NA	NA	NA	NA	NA	1
