# Supplementary material for: Network analysis of pig movements: Loyalty patterns and contact chains of different holding types in Denmark
Source: PLoS One. 2017 Jun 29;12(6):e0179915. doi: 10.1371/journal.pone.0179915 (PMC5491064; doi:10.1371/journal.pone.0179915)
Supplement: S2 File — The file includes supporting tables related to the number of pigs moved per registered pig movement (batch size) and the number of pig movements per holding type: Descriptive statistics of the development of the number of pigs per movement (batch size) of registered pig movements between 1st January 2006 and 31st December 2015 in Denmark (Table 1).Median batch size by holding type of sending and receiving holding of registered pig movements between 1st January 2006 and 31st December 2015 in Denmark (Tables 2 and 3).Average number of pig movements per holding type of sending and receiving holding per year between 1st January 2006 and 31st December 2015 in Denmark (Tables 4 and 5). (PDF) [file pone.0179915.s002.pdf]

S2 File. Number of pigs per movment.

Table 1. Batch sizes. Descriptive statistics of the development of the number of pigs per movment (batch size) of registered pig movements between 1 Jan 2006 and 31 Dec 2015 in Denmark. The column "not available" covers both the number of pig movements without batch sizes and with batch size equal to 0.

	Minimum	1st Quantile	Median	Mean	3rd Quantile	Maximum	Not available
**2006**	1	2	8	49	60	9,780	45,092
**2007**	1	2	9	53	65	9,000	40,811
**2008**	1	3	10	61	76	7,187	38,705
**2009**	1	2	9	67	82	7,035	37,003
**2010**	1	3	10	75	93	7,893	35,843
**2011**	1	3	10	79	100	9,381	37,481
**2012**	1	3	11	83	105	6,918	37,408
**2013**	1	3	11	85	108	7,071	37,769
**2014**	1	3	11	90	115	8,721	40,636
**2015**	1	3	11	96	125	7,588	43,221

Table 2. Median batch size. Median batch size by holding type of sending holding of registered pig movements between 1 Jan 2006 and 31 Dec 2015 in Denmark.

	2006	2007	2008	2009	2010	2011	2012	2013	2014	2015
**Breeding sites**										
Breeding and multiplier herds	10	10	11	11	14	15	15	15	16	19
Quarantine stations	11	10	12	10	8	11	11	11	12	10
Boar stations	3	3	2	2	2	2	2	2	2	2
**Production sites**										
Production herds	8	9	10	9	10	10	11	11	11	11
Weaner herds	60	55	57	29	16	15	15	15	19	20
Free-ranging pig herds	7	7	6	5	5	5	5	5	5	5
Organic pig herds	5	7	8	8	9	8	9	9	7	8
**Hobby sites**										
Hobby herds	3	3	3	3	3	3	4	5	5	3
Pets	3	4	3	147	7	4		4	4	4
Wild boar herds		2		1	2		2	10	6	3
Organic wild boar herds										2
**Transit sites**										
Traders	200	235	52	35	11	22	13	13	38	71
Trade herds		6	20	8	11	10	10	11	13	10
Pig shows	3	3	3	11	15	14	6	5	15	8
Livestock auctions	11	10								
Collection points (CP)	4	4	4	4	6	8	12	13	10	10
Slaughter animal markets	1	5	5					108		7
**Miscellaneous**										
Zoos						2	1	2	2	2
Experimental facilities					2		3			8

**End of production sites**										
Slaughterhouses	3	3	2	4	4	3	2	2	2	2
Export isolation facilities						2	1	2	2	2
CPs for dead animals	3	3	302	4	7	3	2	2	3	3
Cooling stations	2	286	4	1	3	4	4	9	11	10
Rendering plants										

Table 3. Median batch size by holding type of receiving holding of registered pig movements between 1 Jan 2006 and 31 Dec 2015 in Denmark.

	2006	2007	2008	2009	2010	2011	2012	2013	2014	2015
**Breeding sites**										
Breeding and multiplier herds	103	120	120	120	126	147	153	165	169	170
Quarantine stations	7	10	5	12	5	5	4	4	5	4
Boar stations	14	17	16	15	11	13	12	16	19	18
**Production sites**										
Production herds	141	151	180	202	220	240	250	251	255	263
Weaner herds	295	298	325	337	303	401	400	398	396	406
Free-ranging pig herds	119	42	46	115	25	25	20	7	32	41
Organic pig herds	50	85	127	150	188	186	205	228	149	175
**Hobby sites**										
Hobby herds	5	106	75	166	260	6	5	24	6	4
Pets	30	10		324	171	36	2	2	630	2
Wild boar herds		8	2			2	7	58	2	3
Organic wild boar herds										
**Transit sites**										
Traders	44	10	30	28	174	270	250	60	78	53
Trade herds				216	81	14	12	12	12	154
Pig shows	4	4	3	6	2	2	4	2	2	2
Livestock auctions	180	15								
Collection points (CP)	11	15	18	70	73	80	109	151	254	285
Slaughter animal markets	249	116	15						661	634
**Miscellaneous**										
Zoos							1	7	3	3
Experimental facilities			24	22	16	14	14	16	17	10

**End of production sites**										
Slaughterhouses	35	39	44	45	48	52	54	53	58	67
Export isolation facilities							312	65	44	91
CPs for dead animals			325	118	2	220	7	2	4	3
Cooling stations		331					2	1	2	3
Rendering plants	2	2	2	2	2	2	2	2	2	2

Table 4. Average number of pig movements per holding type of sending holding per year between 1 Jan 2006 and 31 Dec 2015 in Denmark.

	2006	2007	2008	2009	2010	2011	2012	2013	2014	2015
**Breeding sites**										
Breeding and multiplier herds	157	167	166	163	148	144	135	137	142	151
Quarantine stations	23	20	17	20	19	20	20	23	27	22
Boar stations	40	43	45	48	47	47	46	44	46	51
**Production sites**										
Production herds	75	80	79	82	87	90	87	88	89	90
Weaner herds	78	81	89	104	108	106	104	102	106	107
Free-ranging pig herds	37	33	33	24	20	19	18	16	18	15
Organic pig herds	32	36	40	36	40	46	49	47	46	52
**Hobby sites**										
Hobby herds	4	10	8	7	4	4	4	5	7	3
Pets	1	1	1	19	8	1		9	1	2
Wild boar herds		1		1	1		6	13	1	3
Organic wild boar herds										3
**Transit sites**										
Traders	5	9	3	16	20	15	8	4	10	10
Trade herds		1	75	70	61	68	72	83	102	66
Pig shows	1	2	1	1	1	1	2	2	1	1
Livestock auctions	215	244								
Collection points (CP)	82	71	93	73	94	67	69	59	57	63
Slaughter animal markets	41	77	112					3		9
**Miscellaneous**										
Zoos						1	1	2	1	2
Experimental facilities					1		18			28

**End of production sites**										
Slaughterhouses	165	87	76	166	180	96	74	80	96	90
Export isolation facilities						3	3	11	12	5
CPs for dead animals	51	1	6	5	3	57	51	23	32	54
Cooling stations	79	41	1	1	4	1	34	7	26	35
Rendering plants										

Table 5. Average number of pig movements per holding type of receiving holding per year between 1 Jan 2006 and 31 Dec 2015 in Denmark.

	2006	2007	2008	2009	2010	2011	2012	2013	2014	2015
**Breeding sites**										
Breeding and multiplier herds	13	15	18	19	20	21	20	21	22	21
Quarantine stations	39	52	19	28	20	24	25	26	29	31
Boar stations	21	19	20	22	22	20	20	17	15	18
**Production sites**										
Production herds	12	14	14	15	16	15	16	16	17	17
Weaner herds	24	29	29	25	23	22	21	21	23	24
Free-ranging pig herds	6	6	6	6	4	3	2	3	6	2
Organic pig herds	3	3	4	5	4	4	5	5	8	7
**Hobby sites**										
Hobby herds	1	4	9	4	9	2	3	5	6	1
Pets	2	1		12	4	2	1	1	4	1
Wild boar herds		1	1			2	1	4	1	2
Organic wild boar herds										
**Transit sites**										
Traders	15	6	5	6	25	93	24	5	8	52
Trade herds				14	21	41	62	81	78	22
Pig shows	1	2	1	2	2	2	2	3	3	2
Livestock auctions	539	585								
Collection points (CP)	2,804	2,467	1,892	1,445	1,319	1,451	1,183	1,149	834	836
Slaughter animal markets	217	512	1,299						1	2
**Miscellaneous**										
Zoos							1	1	2	2
Experimental facilities			9	16	24	23	25	52	26	51

**End of production sites**										
Slaughterhouses	4,272	4,095	3,858	3,682	3,665	3,752	3,498	3,419	3,074	2,797
Export isolation facilities							3	2	12	4
CPs for dead animals			8	1	1	5	43	48	27	55
Cooling stations		40					23	38	64	86
Rendering plants	401,386	405,473	370,127	350,848	340,541	333,422	302,656	295,889	297,428	295,306
