# Supplementary material for: Network analysis of pig movements: Loyalty patterns and contact chains of different holding types in Denmark
Source: PLoS One. 2017 Jun 29;12(6):e0179915. doi: 10.1371/journal.pone.0179915 (PMC5491064; doi:10.1371/journal.pone.0179915)
Supplement: S3 File — The file includes supporting figures and tables related to the in- and out-loyalty of the investigated holding types: In-loyalty for each pair of consecutive years for the whole network of pig movements from 1st January 2006 to 31st December 2015 in Denmark (Figure 1),Out-loyalty for each pair of consecutive years for the whole network of pig movements from 1st January 2006 to 31st December 2015 in Denmark (Figure 2).In- and out-loyalty for (a) breeding sites, (b) production sites, (c) hobby sites, (d) transit sites, (e) miscellaneous sites, and (f) end of production sites (Figures 3–8),Descriptive summaries of in- and out-loyalty per holding type (Tables 1 and 2). (PDF) [file pone.0179915.s003.pdf]

S2 File. In- and out-loyalty per holding type.

Figure 1. In-loyalty. In-loyalty for each pair of consecutive years for the whole network of pig movements from 1 Jan 2006 to 31 Dec 2015 in Denmark.

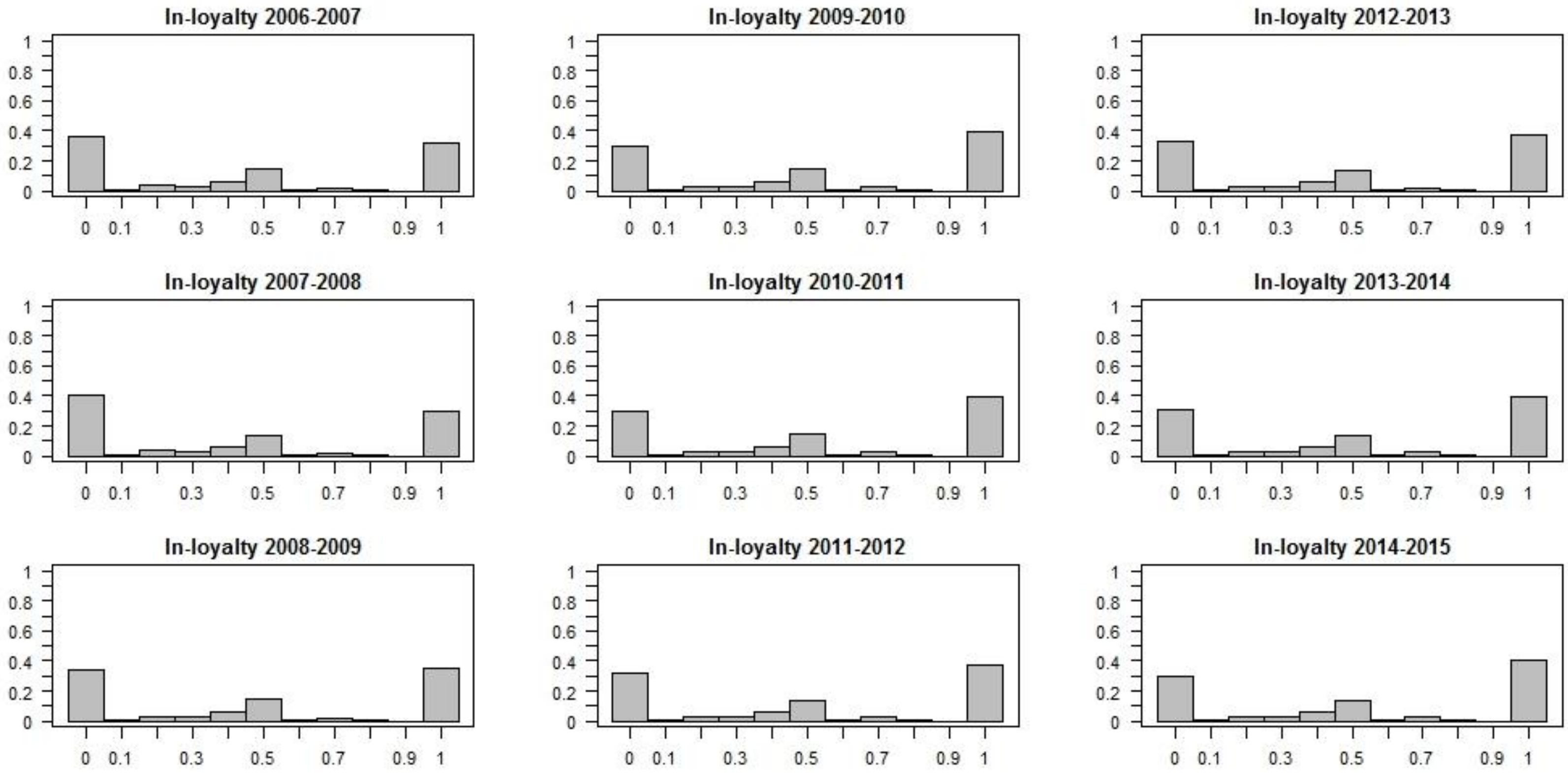

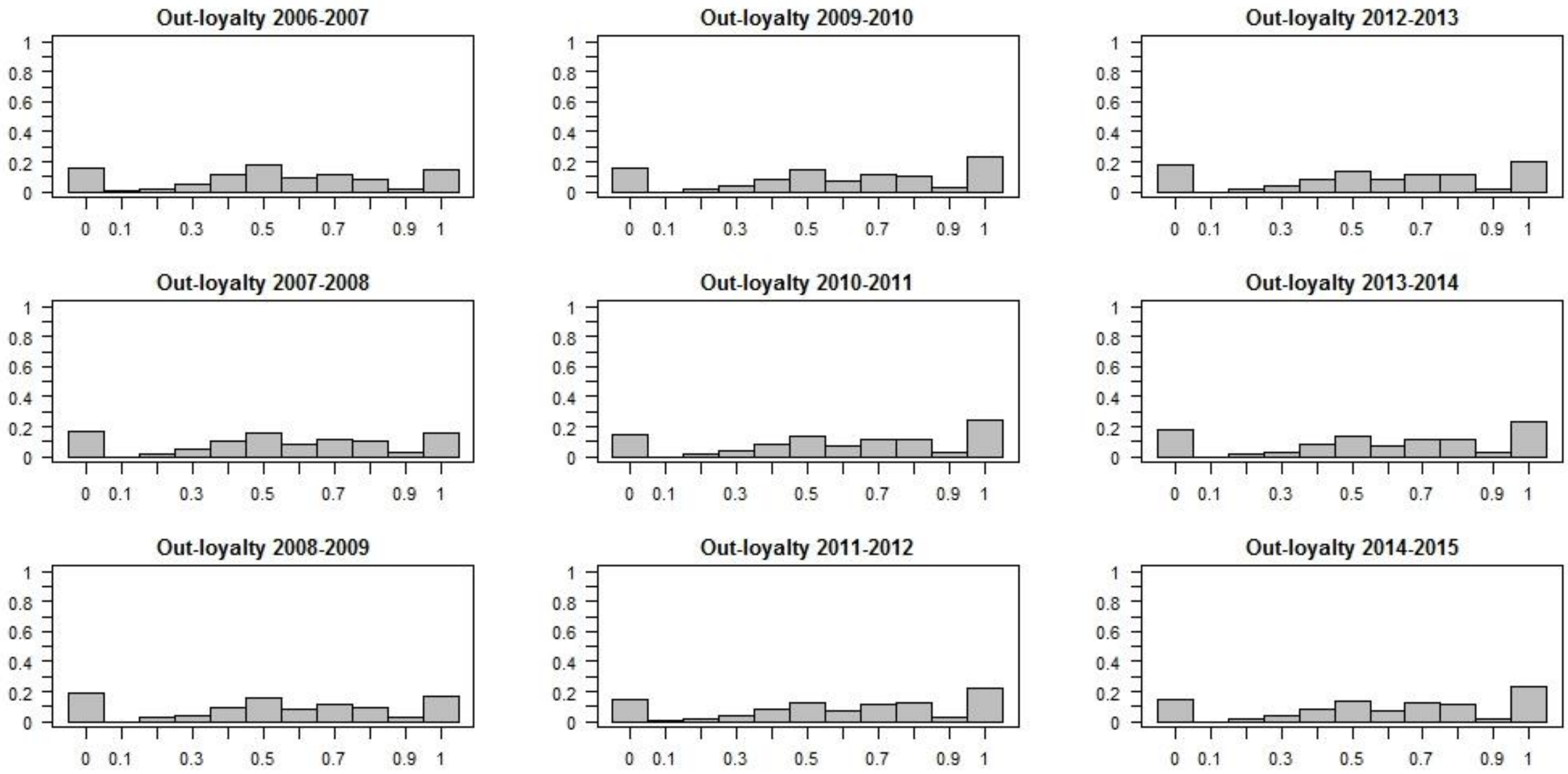

Figure 2. Out-loyalty. Out-loyalty for each pair of consecutive years for the whole network of pig movements from 1 Jan 2006 to 31 Dec 2015 in Denmark.

Figure 3. In- and out-loyalty for breeding sites.

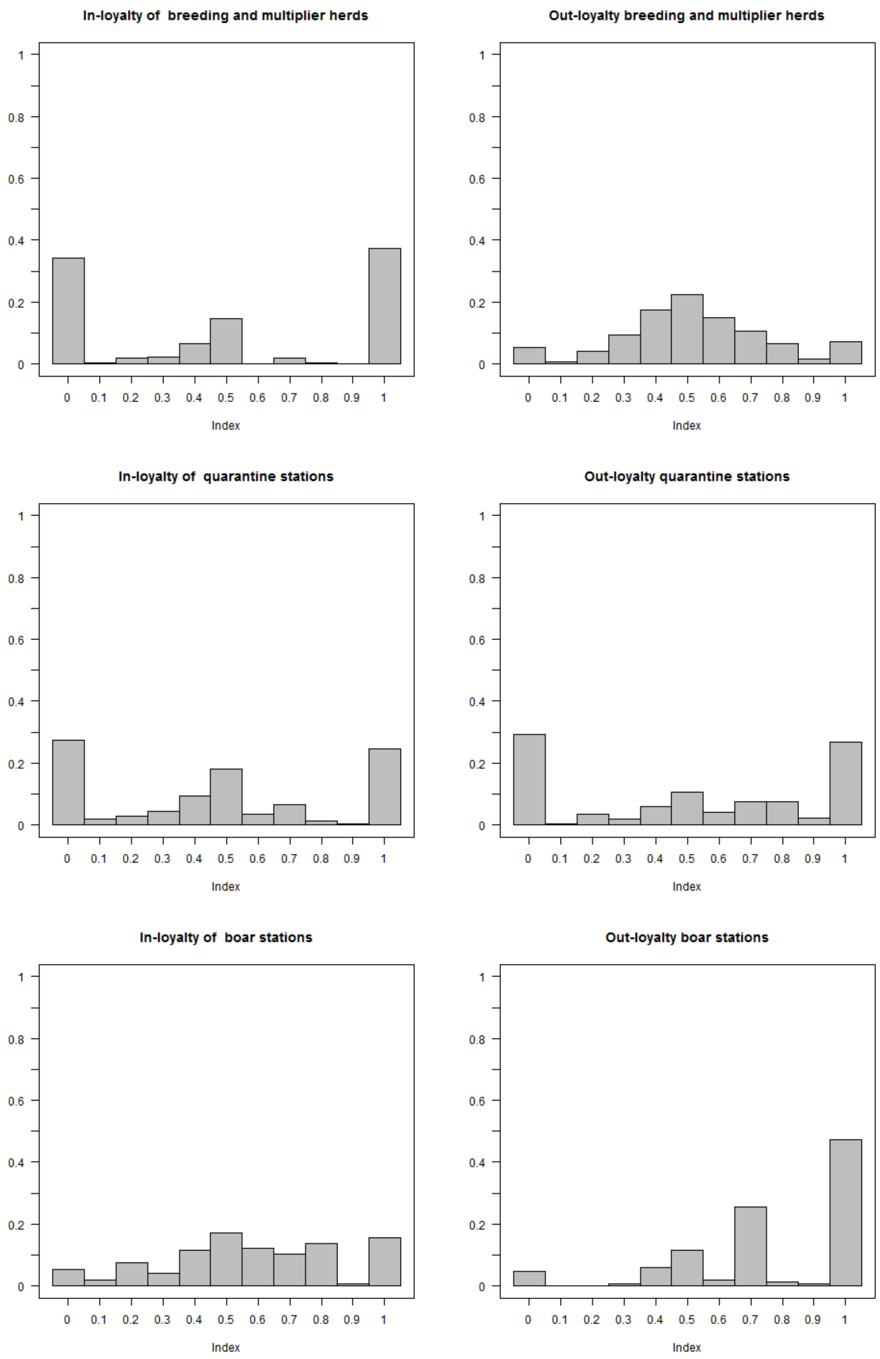

Figure 4. In- and out-loyalty for production sites.

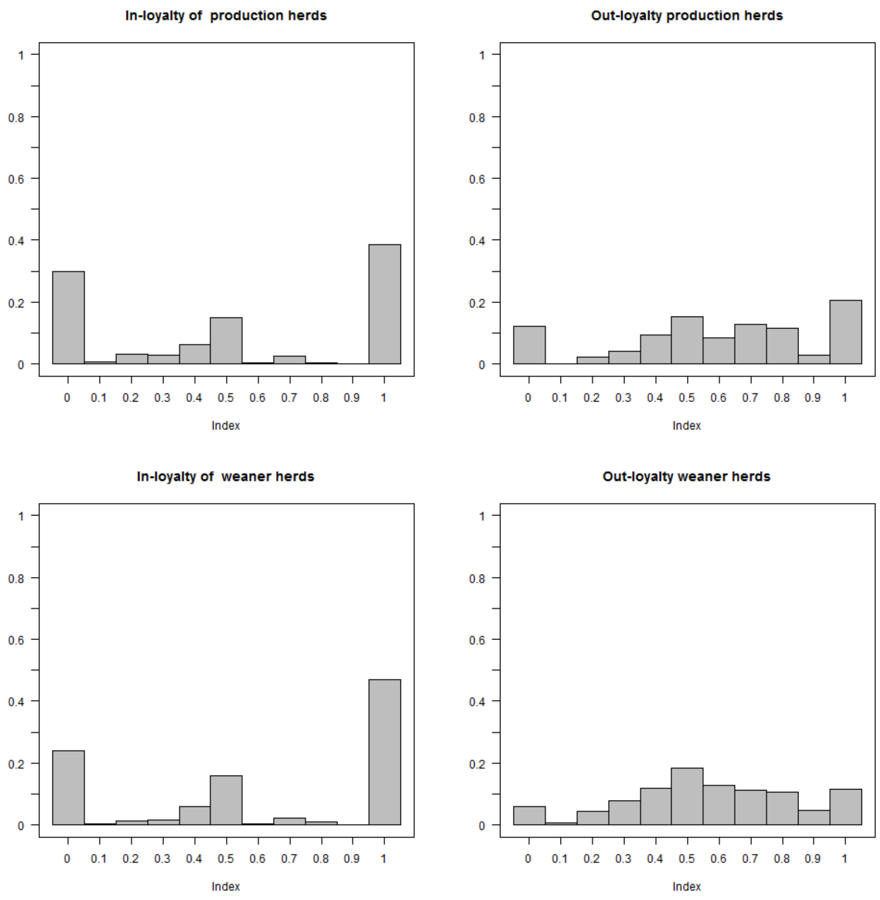

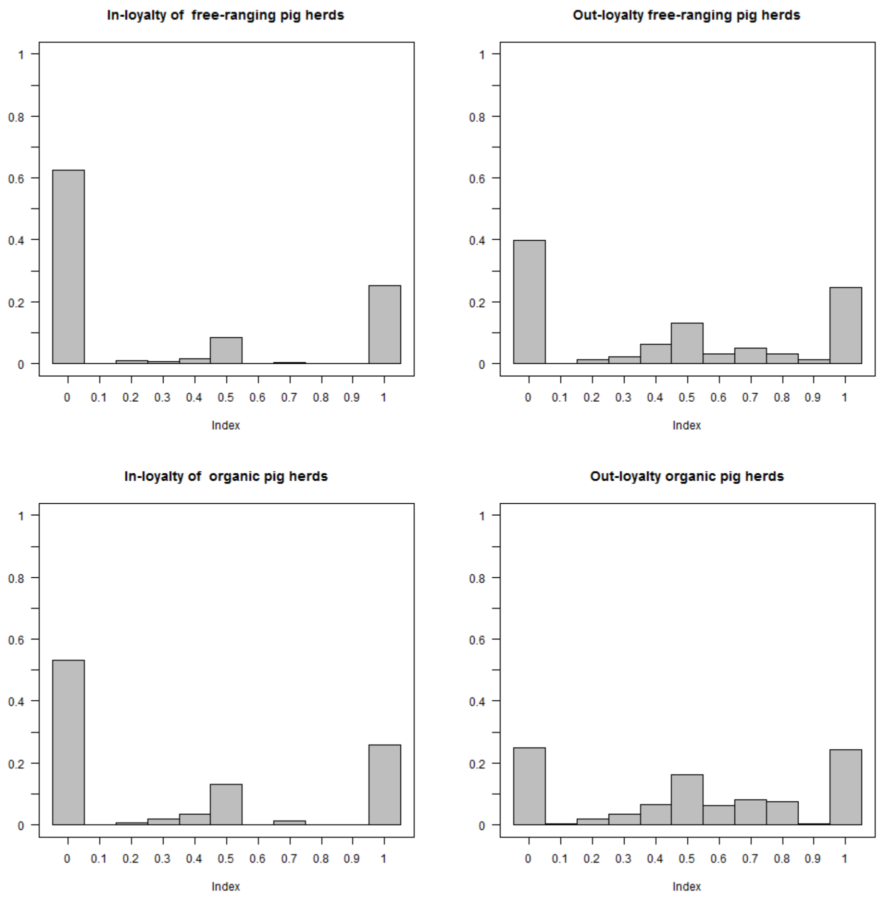

In-loyalty of free-ranging pig herds
Out-loyalty free-ranging pig herds
In-loyalty of organic pig herds
Out-loyalty organic pig herds
0
0.2
0.4
0.6
0.8
1
0.1
0.3
0.5
0.7
0.9
Index

Figure 5. In- and out-loyalty for hobby sites.

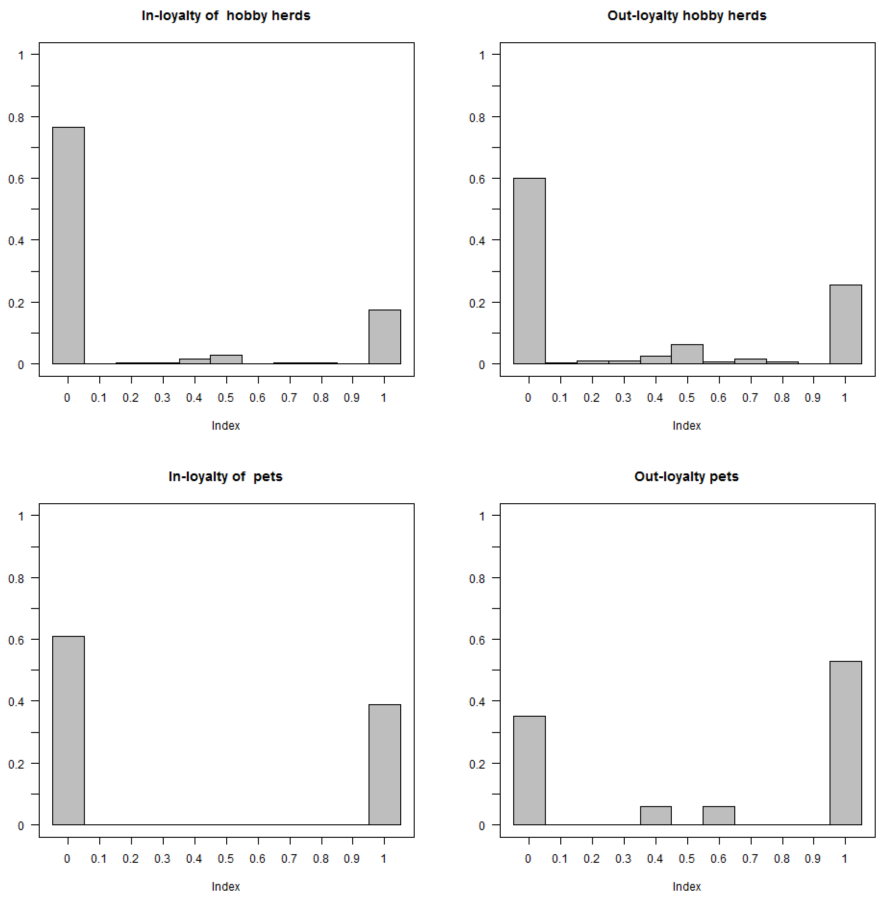

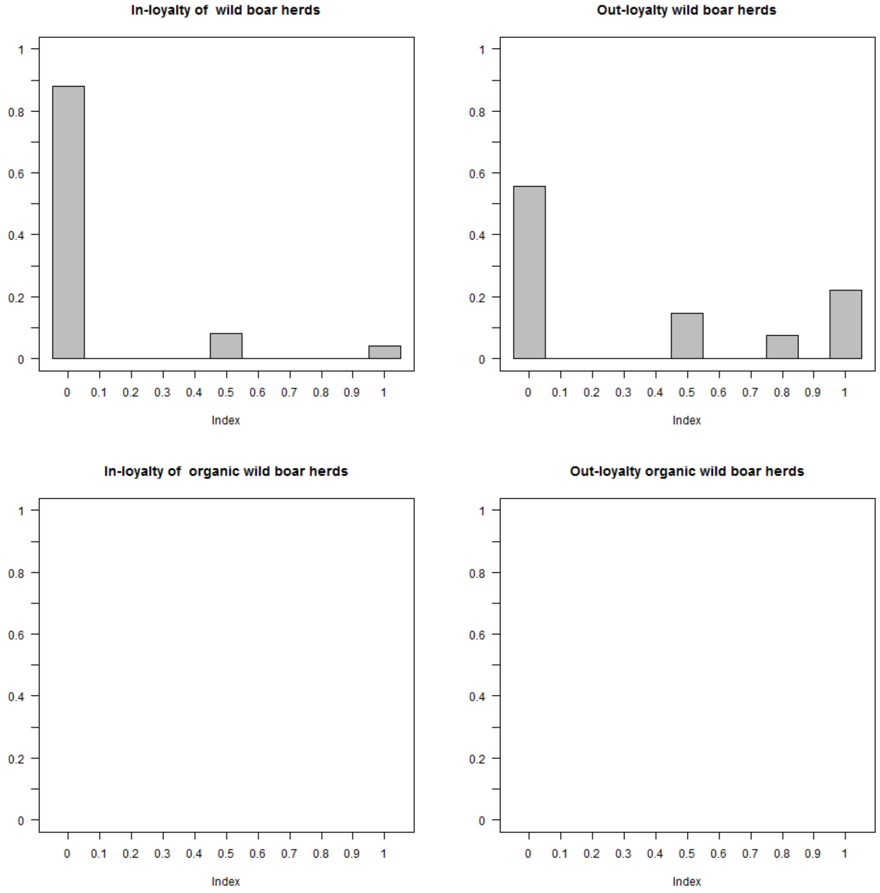
In-loyalty of wild boar herds
Out-loyalty wild boar herds
In-loyalty of organic wild boar herds
Out-loyalty organic wild boar herds
0
0.2
0.4
0.6
0.8
1
0.1
0.3
0.5
0.7
0.9
Index

Figure 6. In- and out-loyalty for transit sites.

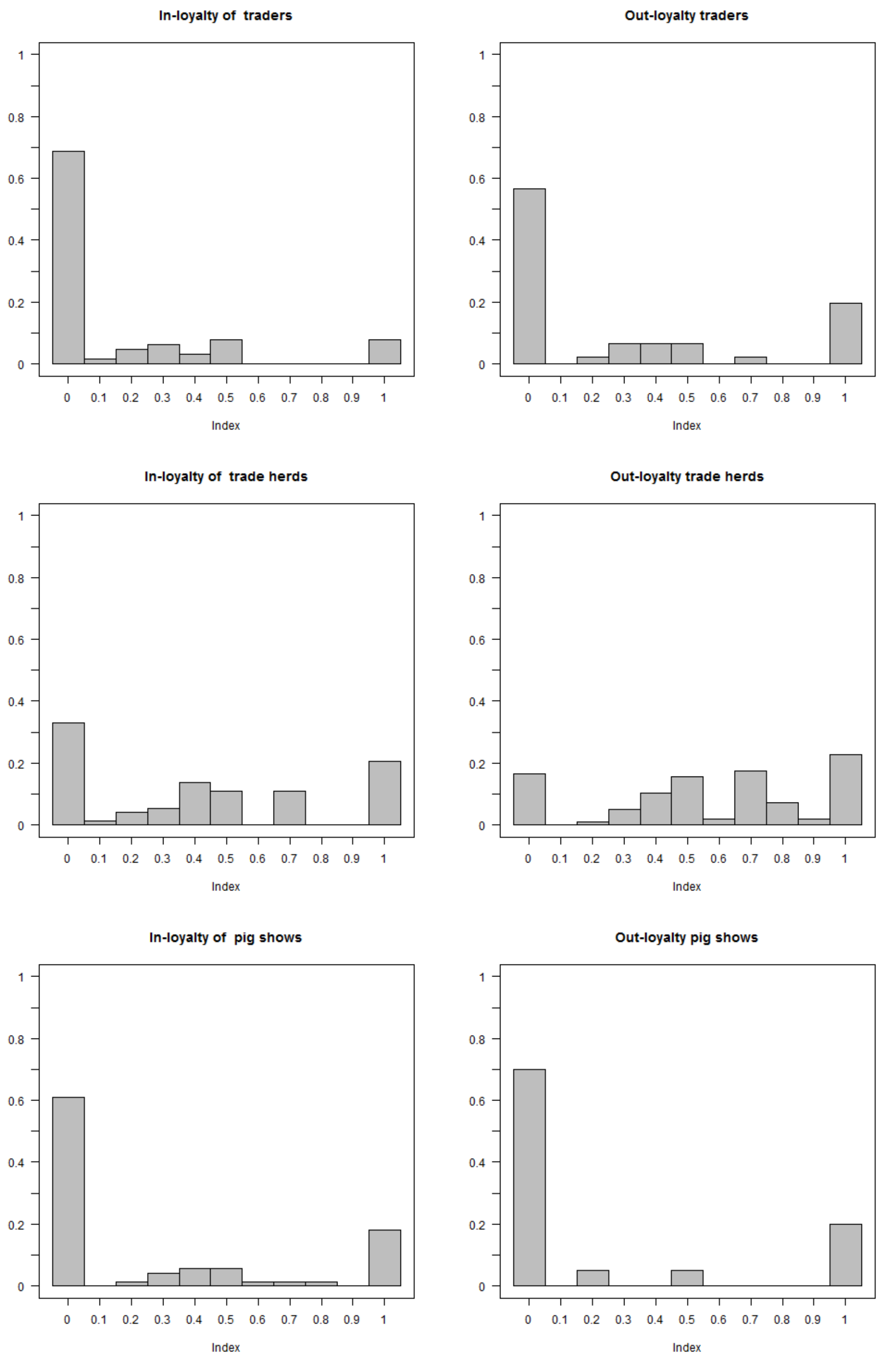

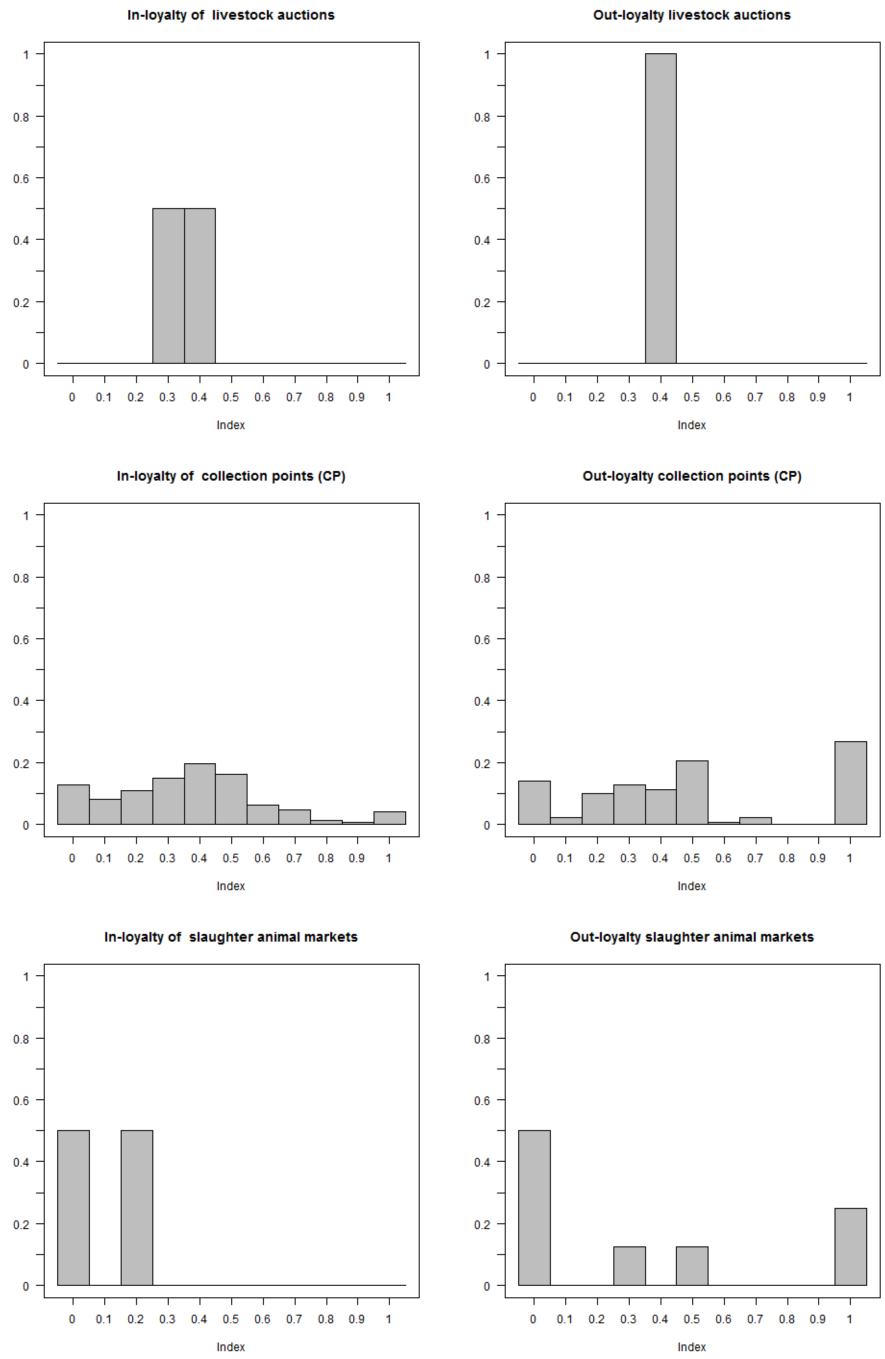
In-loyalty of livestock auctions
Out-loyalty livestock auctions
In-loyalty of collection points (CP)
Out-loyalty collection points (CP)
In-loyalty of slaughter animal markets
Out-loyalty slaughter animal markets
0 0.2 0.4 0.6 0.8 1
0 0.1 0.2 0.3 0.4 0.5 0.6 0.7 0.8 0.9 1
Index

Figure 7. In- and out-loyalty for miscellaneous sites.

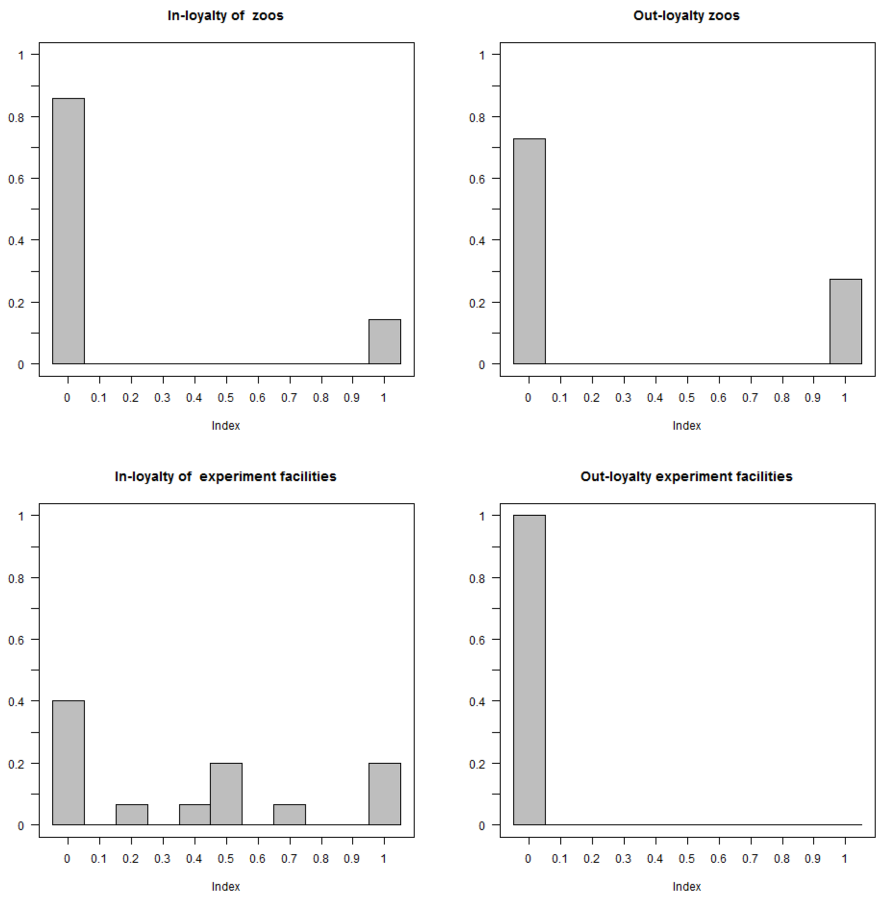

Figure 8. In- and out-loyalty for end of production sites.

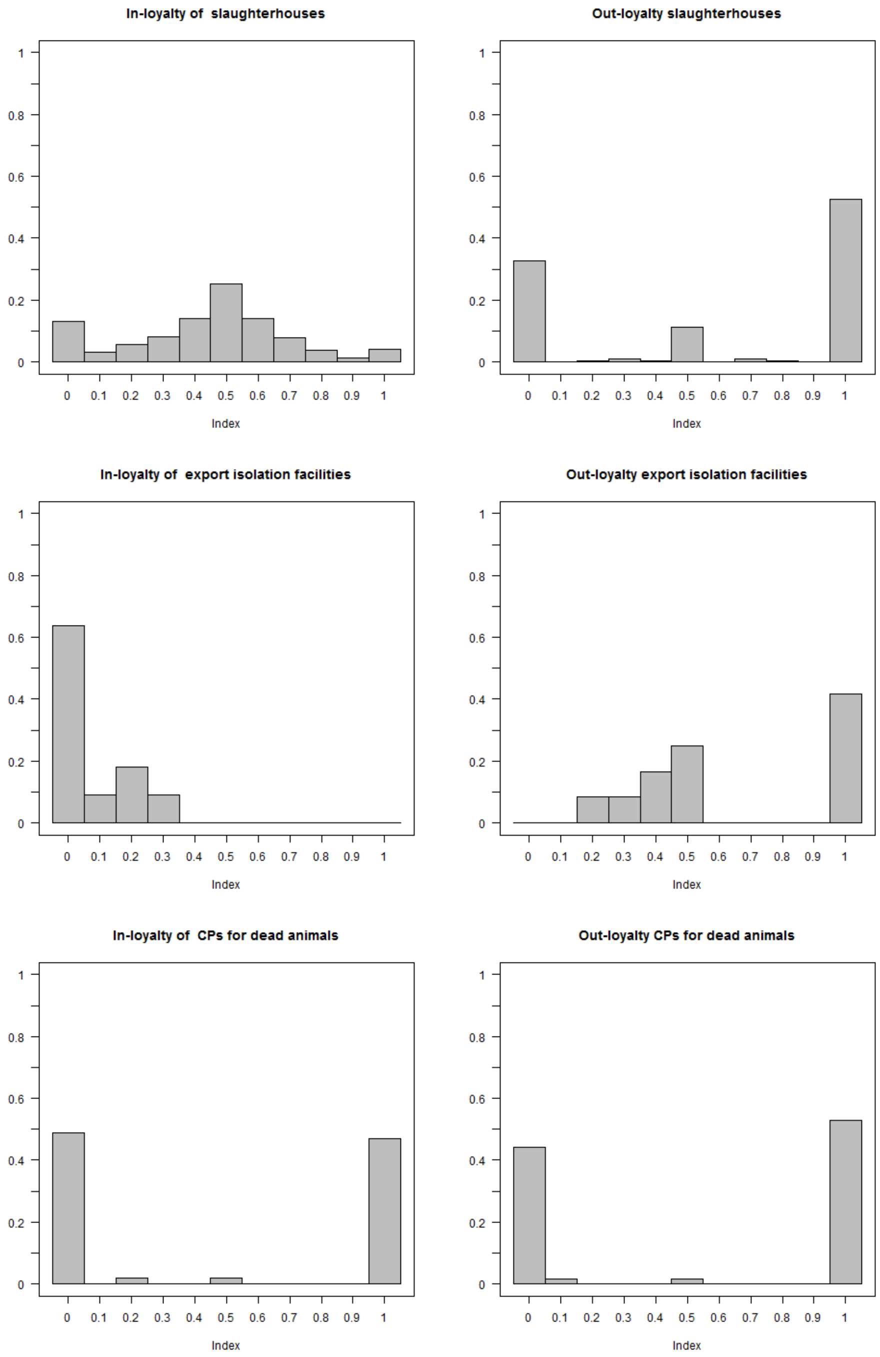

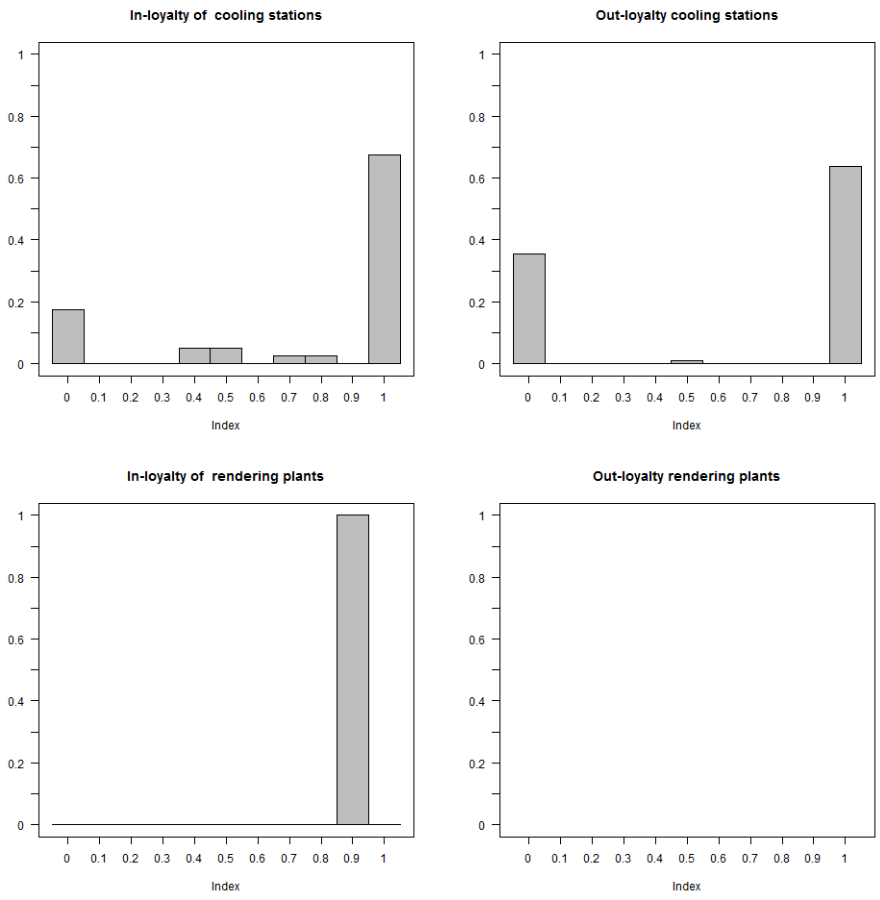

In-loyalty of cooling stations
Out-loyalty cooling stations
In-loyalty of rendering plants
Out-loyalty rendering plants
Index

Table 1. Summary of in-loyalty per holding type. Levels for in-loyalty: (1) low – mean < 0.45, (2) intermediate – 0.45 ≤ mean ≤ 0.55 and (3) high – mean > 0.55

	Minimum	1st quantile	Median	Mean	3rd quantile	Maximum	Level
**Breeding sites**							
Breeding and multiplier herds	0,00	0,00	0,50	0,50	1,00	1,00	intermediate
Quarantine stations	0,00	0,00	0,49	0,46	0,78	1,00	intermediate
Boar stations	0,00	0,38	0,58	0,55	0,75	1,00	intermediate
**Production sites**							
Production herds	0,00	0,00	0,50	0,52	1,00	1,00	intermediate
Weaner herds	0,00	0,17	0,67	0,60	1,00	1,00	high
Free-ranging pig herds	0,00	0,00	0,00	0,31	1,00	1,00	low
Organic pig herds	0,00	0,00	0,00	0,35	1,00	1,00	low
**Hobby sites**							
Hobby herds	0,00	0,00	0,00	0,20	0,00	1,00	low
Pets	0,00	0,00	0,00	0,39	1,00	1,00	low
Wild boar herds	0,00	0,00	0,00	0,08	0,00	1,00	low
Organic wild boar herds	NA	NA	NA	NA	NA	NA	not available
**Transit sites**							
Traders	0,00	0,00	0,00	0,15	0,20	1,00	low
Trade herds	0,00	0,00	0,35	0,40	0,67	1,00	low
Pig shows	0,00	0,00	0,00	0,27	0,50	1,00	low
Livestock auctions	0,25	0,27	0,29	0,29	0,30	0,32	low
Collection points (CP)	0,00	0,13	0,33	0,32	0,44	1,00	low
Slaughter animal markets	0,00	0,00	0,05	0,06	0,12	0,14	low
**Miscellaneous**							
Zoos	0,00	0,00	0,00	0,14	0,00	1,00	low
Experimental facilities	0,00	0,00	0,33	0,38	0,58	1,00	low
**End of production sites**							

Slaughterhouses	0,00	0,27	0,43	0,40	0,54	1,00	low
Export isolation facilities	0,00	0,00	0,00	0,06	0,11	0,29	low
CPs for dead animals	0,00	0,00	0,18	0,48	1,00	1,00	intermediate
Cooling stations	0,00	0,50	1,00	0,75	1,00	1,00	high
Rendering plants	0,81	0,85	0,86	0,86	0,87	0,89	high

Table 2. Summary of out-loyalty per holding type. Levels for out-loyalty: (1) low – mean < 0.45, (2) intermediate – 0.45 ≤ mean ≤ 0.55 and (3) high – mean > 0.55

	Minimum	1st quantile	Median	Mean	3rd quantile	Maximum	
**Breeding sites**							
Breeding and multiplier herds	0,00	0,33	0,49	0,49	0,62	1,00	intermediate
Quarantine stations	0,00	0,00	0,50	0,50	1,00	1,00	intermediate
Boar stations	0,00	0,63	0,67	0,75	1,00	1,00	high
**Production sites**							
Production herds	0,00	0,38	0,60	0,58	0,80	1,00	high
Weaner herds	0,00	0,36	0,54	0,54	0,71	1,00	intermediate
Free-ranging pig herds	0,00	0,00	0,43	0,43	0,83	1,00	low
Organic pig herds	0,00	0,08	0,50	0,51	0,80	1,00	intermediate
**Hobby sites**							
Hobby herds	0,00	0,00	0,00	0,32	1,00	1,00	low
Pets	0,00	0,00	1,00	0,58	1,00	1,00	high
Wild boar herds	0,00	0,00	0,00	0,35	0,75	1,00	low
Organic wild boar herds	NA	NA	NA	NA	NA	NA	not available
**Transit sites**							
Traders	0,00	0,00	0,00	0,29	0,50	1,00	low
Trade herds	0,00	0,35	0,60	0,56	0,80	1,00	high
Pig shows	0,00	0,00	0,00	0,24	0,28	1,00	low
Livestock auctions	0,36	0,36	0,37	0,37	0,37	0,38	low
Collection points (CP)	0,00	0,20	0,41	0,47	1,00	1,00	intermediate
Slaughter animal markets	0,00	0,00	0,13	0,34	0,63	1,00	low
**Miscellaneous**							
Zoos	0,00	0,00	0,00	0,27	0,50	1,00	low
Experimental facilities	0,00	0,00	0,00	0,00	0,00	0,00	low
**End of production sites**							

Slaughterhouses	0,00	0,00	1,00	0,60	1,00	1,00	high
Export isolation facilities	0,20	0,38	0,46	0,63	1,00	1,00	high
CPs for dead animals	0,00	0,00	1,00	0,54	1,00	1,00	intermediate
Cooling stations	0,00	0,00	1,00	0,64	1,00	1,00	high
Rendering plants	NA	NA	NA	NA	NA	NA	not available
