# Supplementary material for: Network analysis of pig movements: Loyalty patterns and contact chains of different holding types in Denmark
Source: PLoS One. 2017 Jun 29;12(6):e0179915. doi: 10.1371/journal.pone.0179915 (PMC5491064; doi:10.1371/journal.pone.0179915)
Supplement: S4 File — The file includes supporting figures and tables related to the in-going and out-going contact chains: Size of (a) in-going and (b) out-going contact chain for the whole pig movement network in Denmark from 1st January 2006 to 31st December 2015 (Figure 1).In-going and out-going contact chains for (a) breeding sites, (b) production sites, (c) hobby sites, (d) transit sites, (e) miscellaneous sites, and (f) end of production sites (Figures 2–7). (PDF) [file pone.0179915.s004.pdf]

S4 File. Ingoing- and outgoing contact chains.

Figure 1. Contact chains. Size of (a) in-going and (b) out-going contact chain for the whole pig movement network in Denmark from 1 Jan 2006 to 31 Dec 2015.

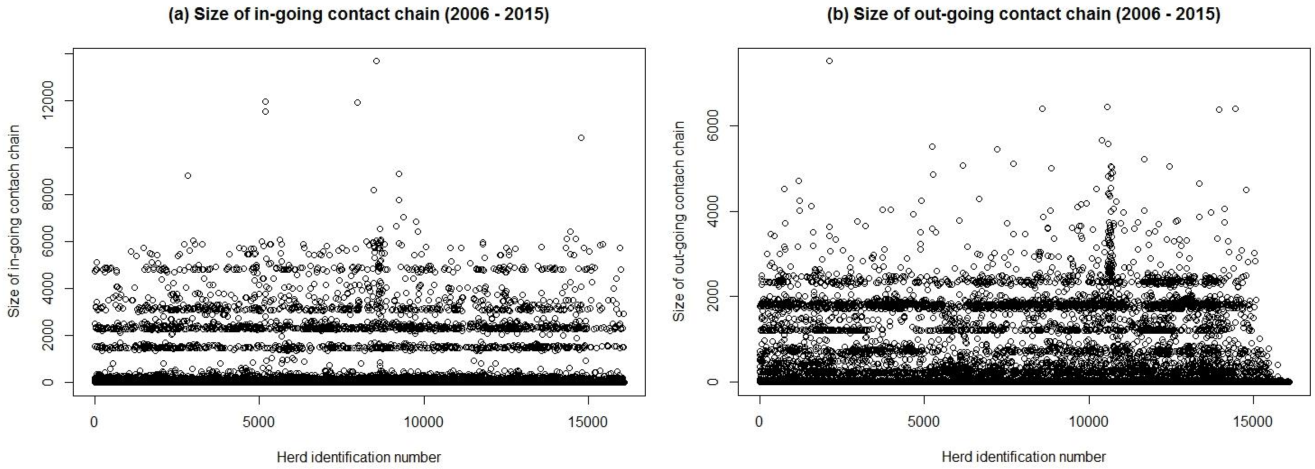

Figure 2. In-going and out-going contact chains for breeding sites.

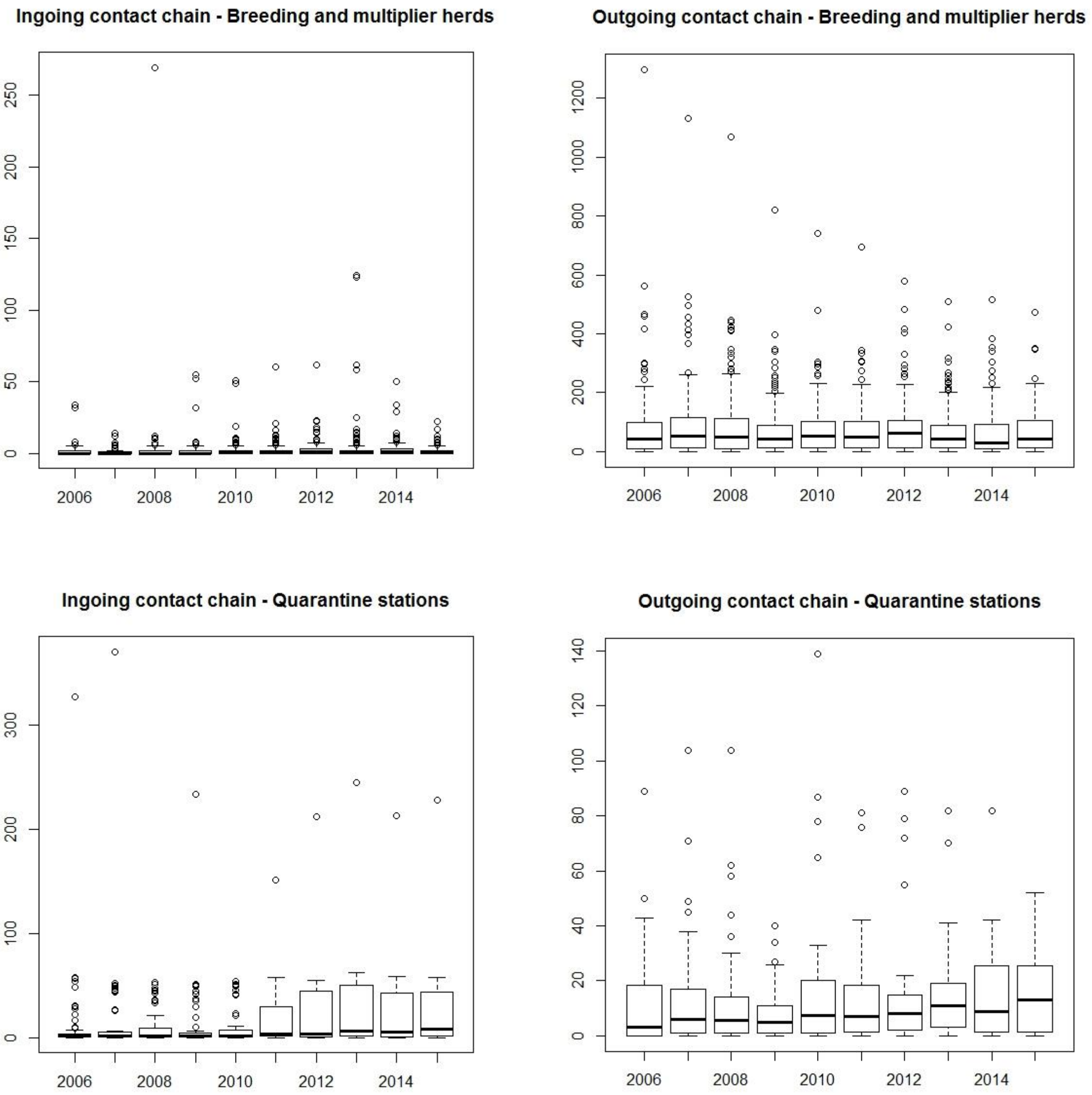

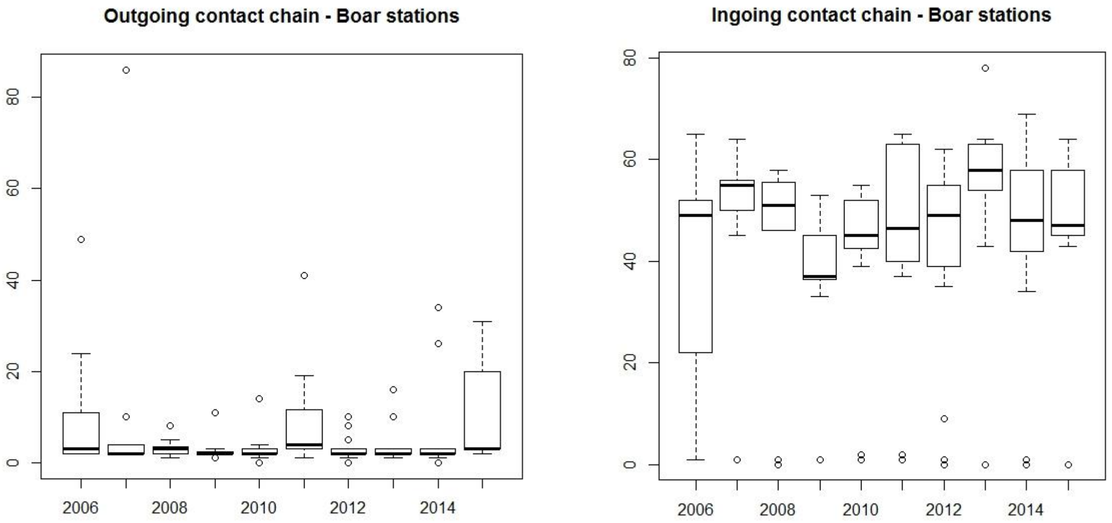

Outgoing contact chain - Boar stations
0
20
40
60
80
2006
2008
2010
2012
2014
Ingoing contact chain - Boar stations
0
20
40
60
80
2006
2008
2010
2012
2014

Figure 3. In-going and out-going contact chains for production sites.

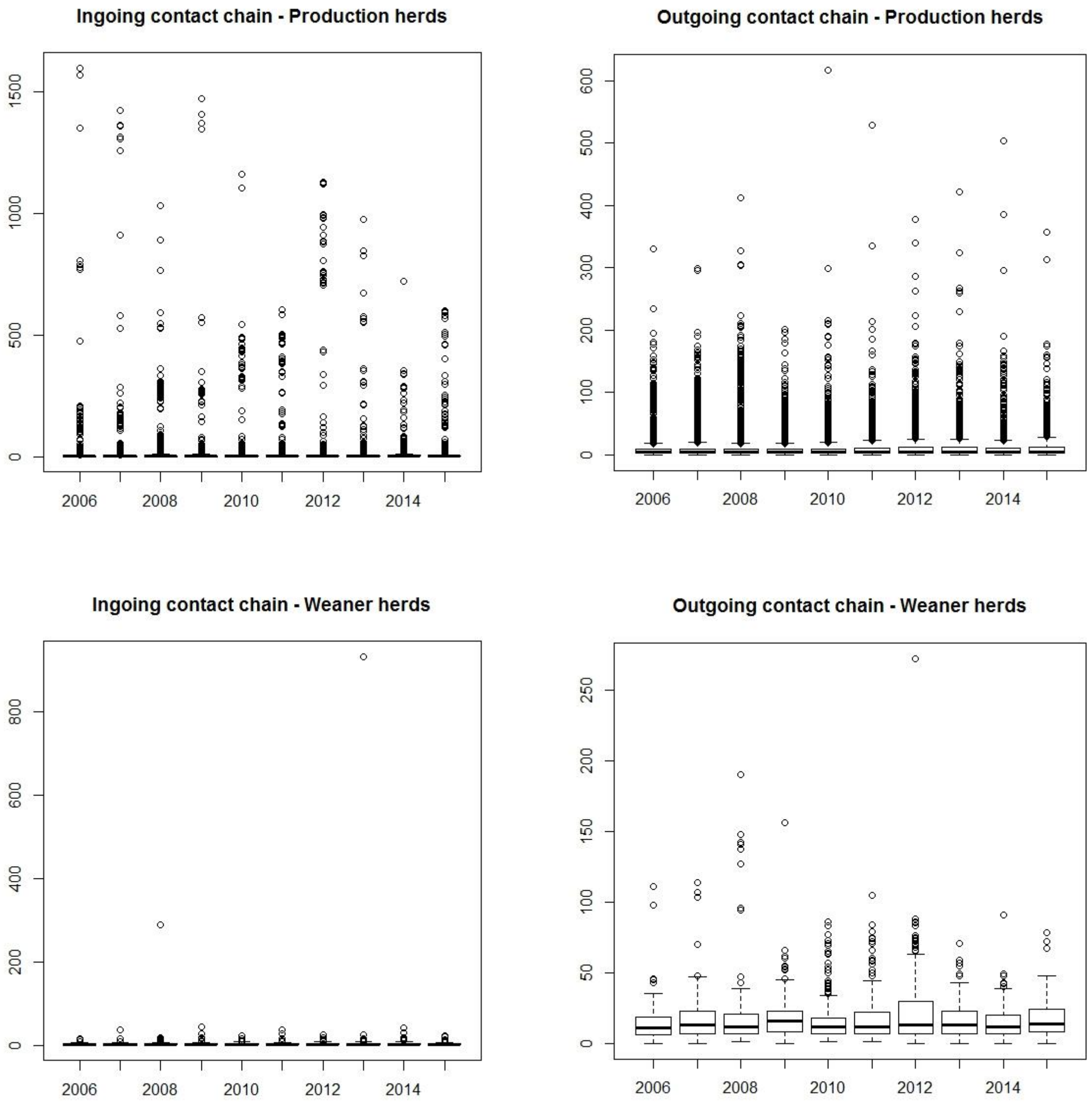

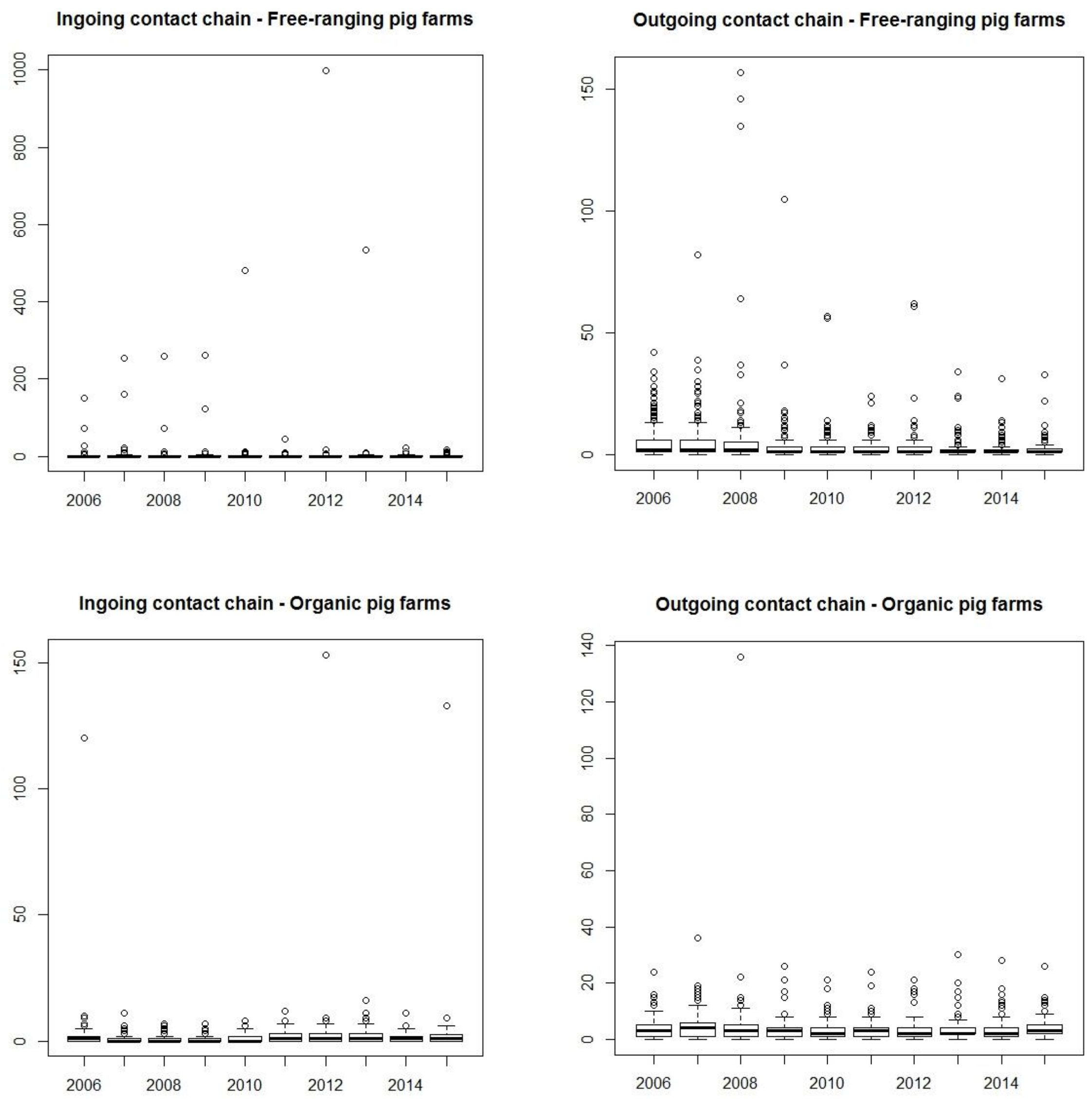
Ingoing contact chain - Free-ranging pig farms
0
200
400
600
800
1000
2006
2008
2010
2012
2014
Outgoing contact chain - Free-ranging pig farms
0
50
100
150
2006
2008
2010
2012
2014
Ingoing contact chain - Organic pig farms
0
50
100
150
2006
2008
2010
2012
2014
Outgoing contact chain - Organic pig farms
0
20
40
60
80
100
120
140
2006
2008
2010
2012
2014

Figure 4. In-going and out-going contact chains for hobby sites.

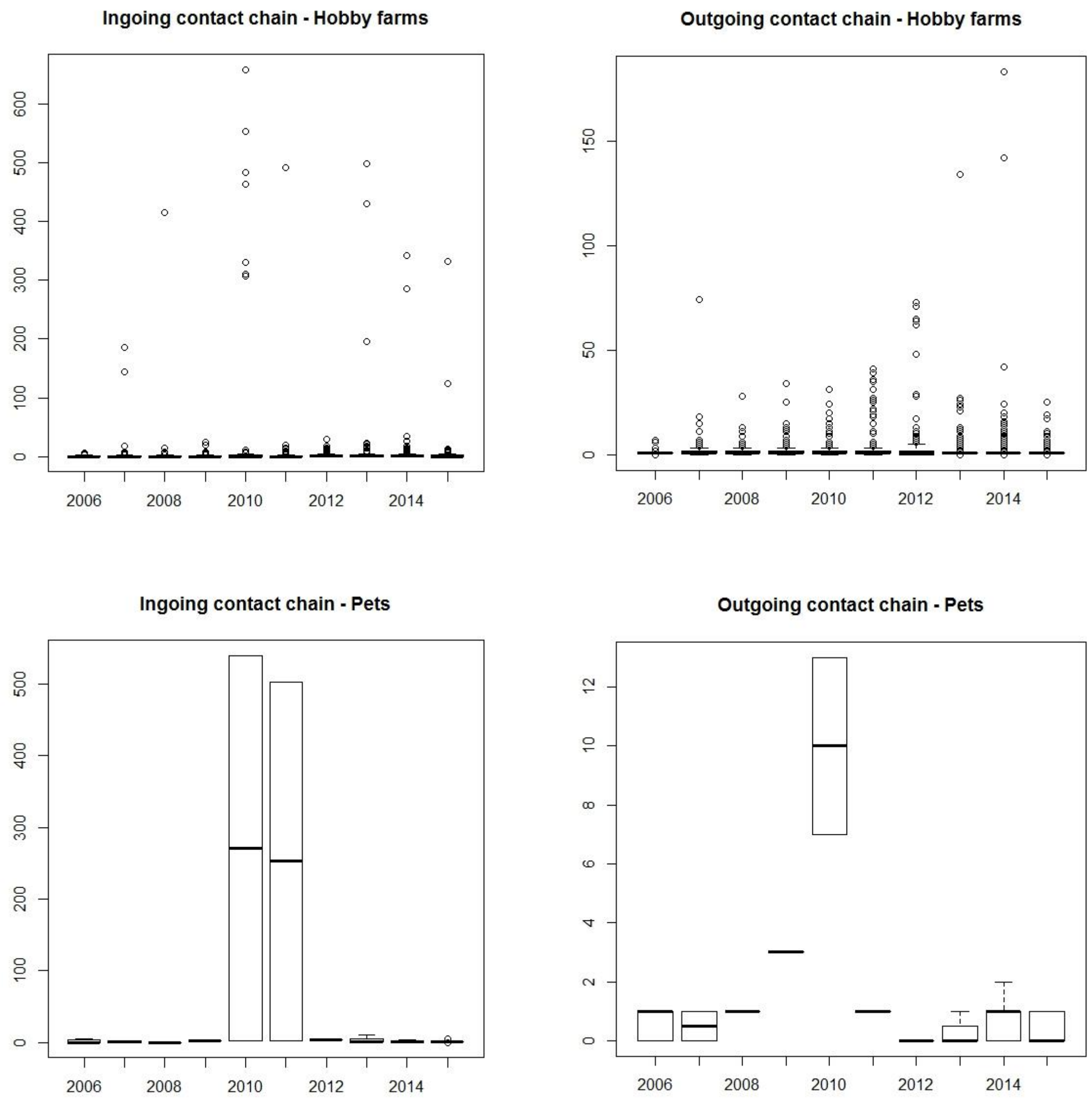

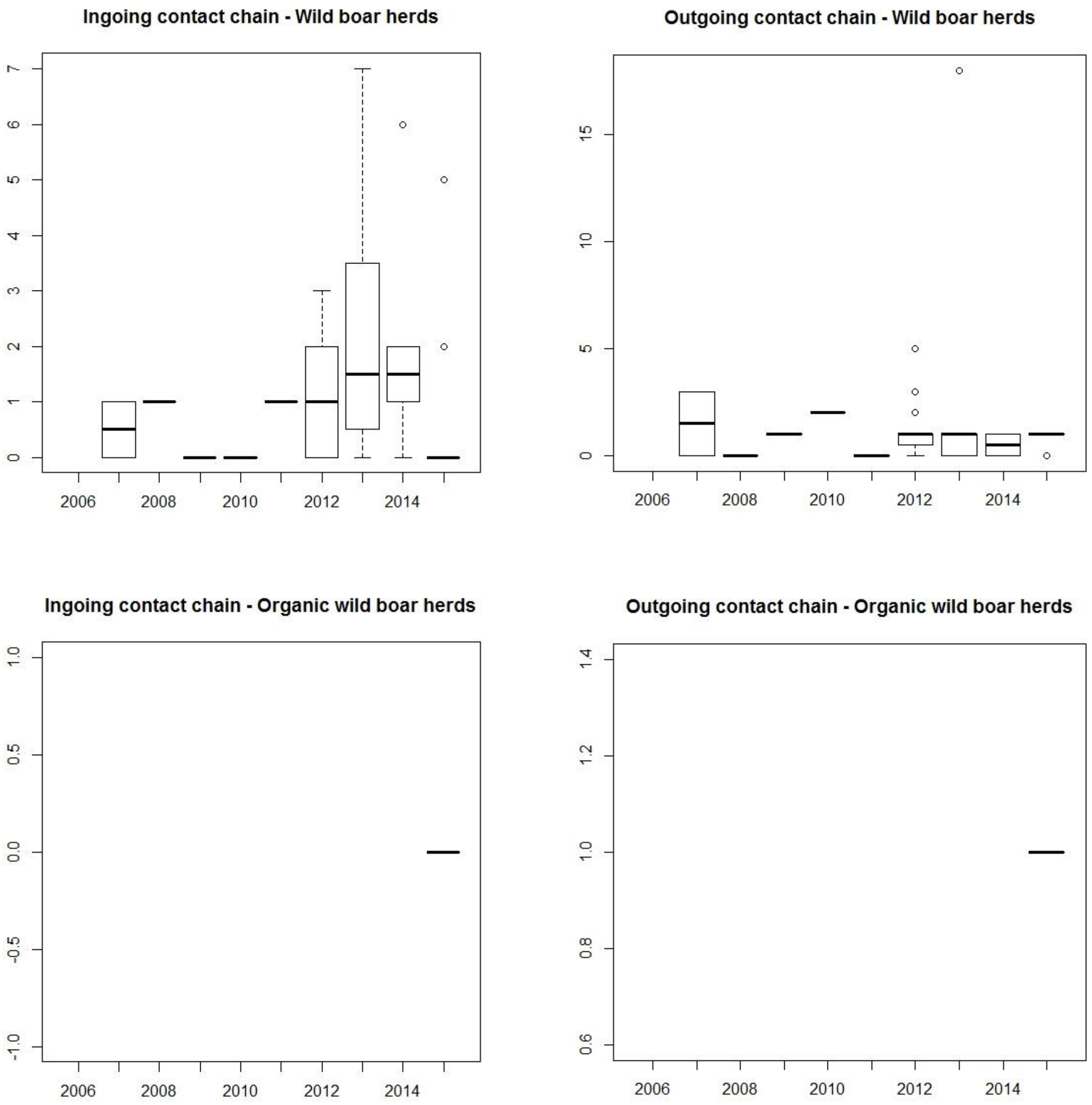

Ingoing contact chain - Wild boar herds
Outgoing contact chain - Wild boar herds
Ingoing contact chain - Organic wild boar herds
Outgoing contact chain - Organic wild boar herds
2006
2008
2010
2012
2014

Figure 5. In-going and out-going contact chains for transit sites.

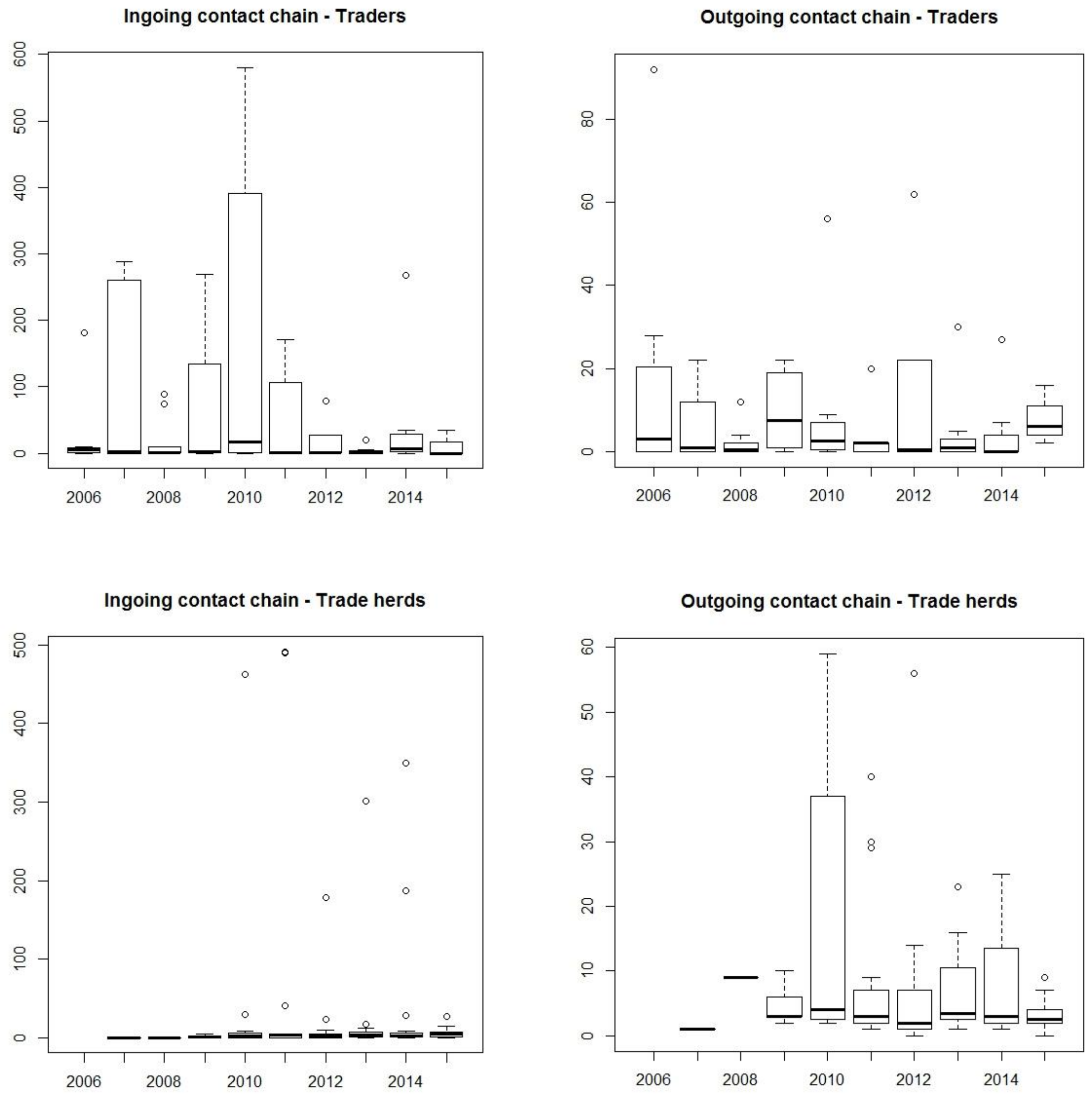

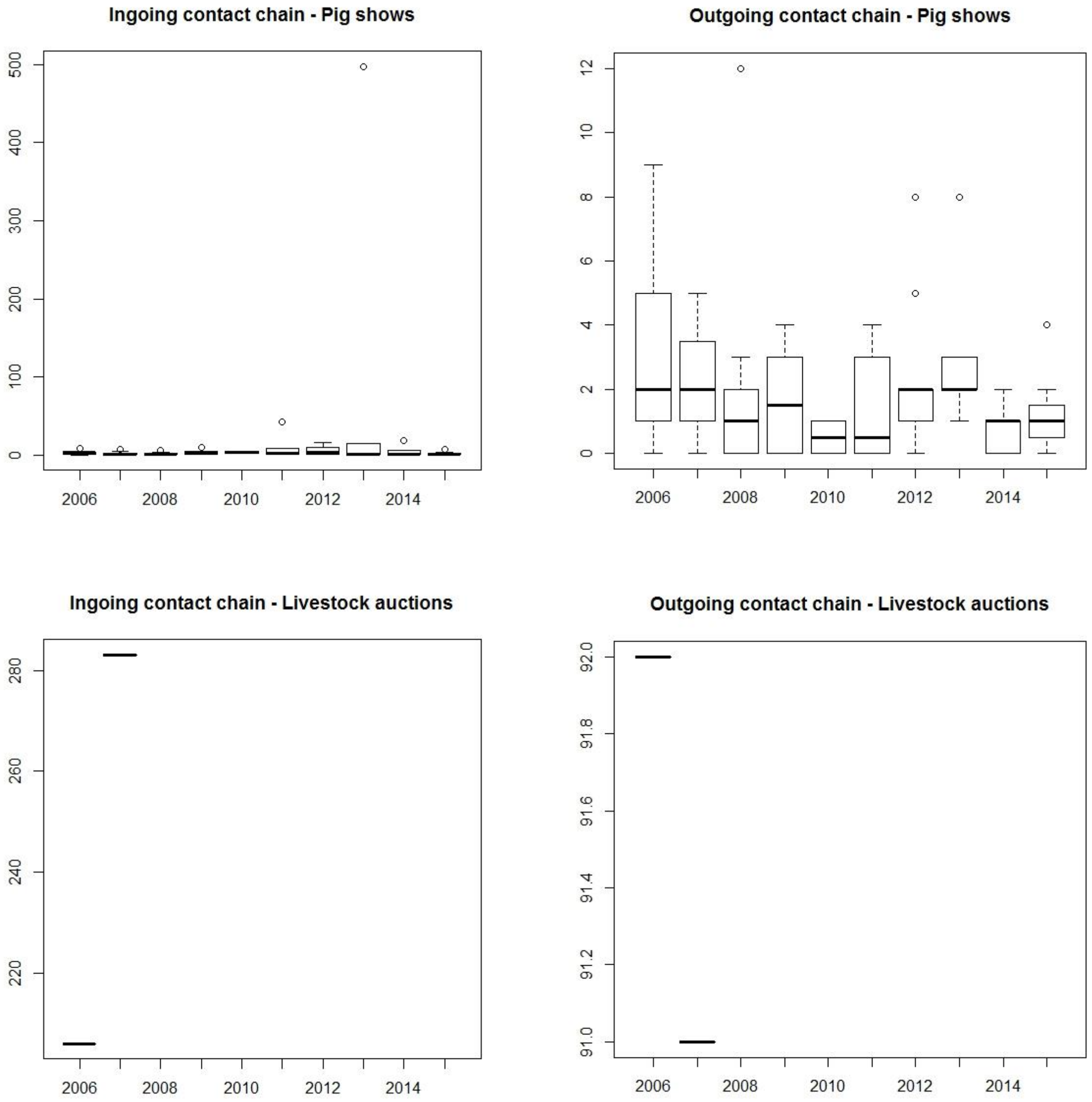

Ingoing contact chain - Pig shows
Outgoing contact chain - Pig shows
Ingoing contact chain - Livestock auctions
Outgoing contact chain - Livestock auctions
0
100
200
300
400
500
0
2
4
6
8
10
12
220
240
260
280
91.0
91.2
91.4
91.6
91.8
92.0
2006
2008
2010
2012
2014

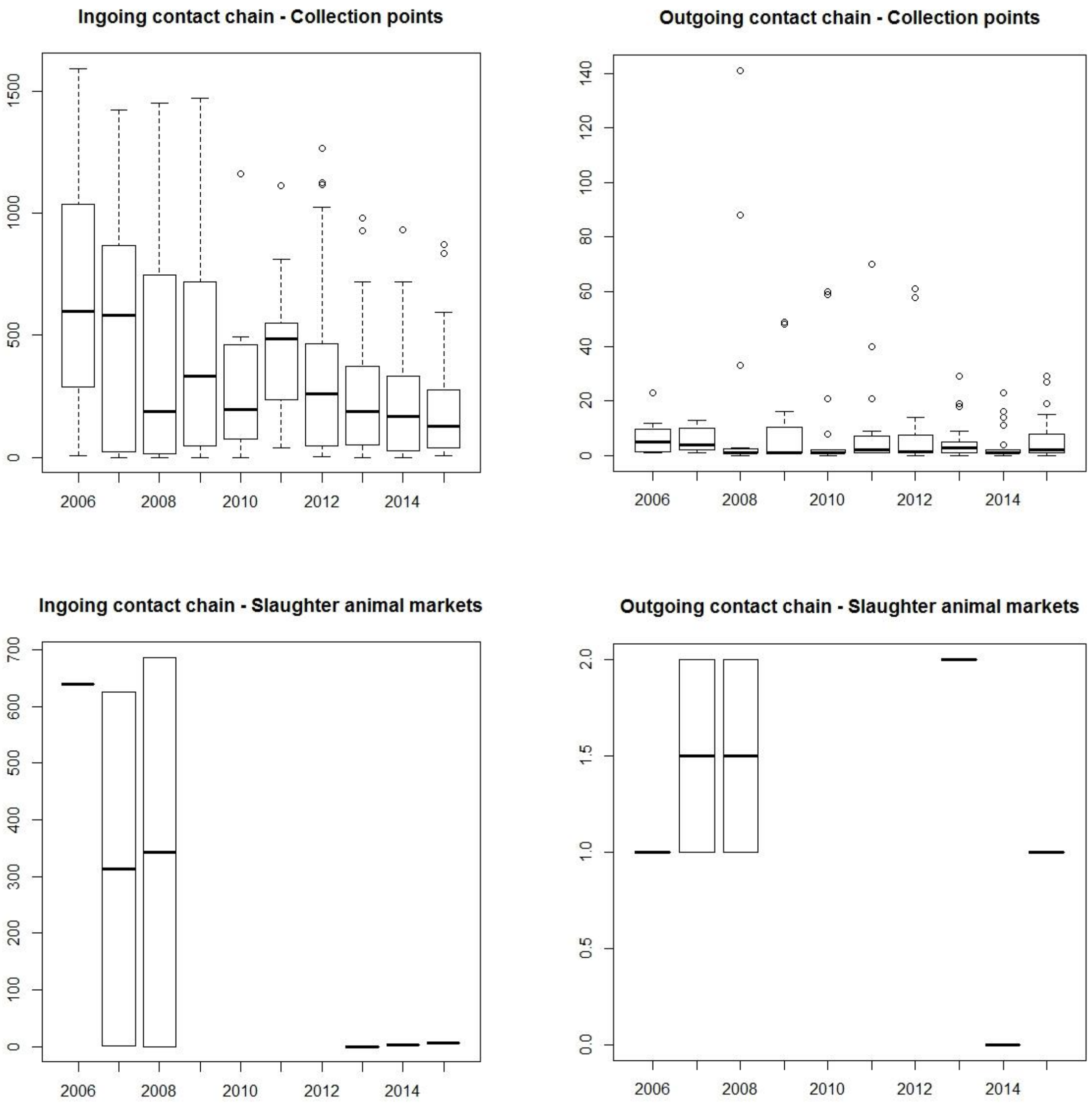
Ingoing contact chain - Collection points
0
500
1000
1500
2006
2008
2010
2012
2014
Outgoing contact chain - Collection points
0
20
40
60
80
100
120
140
2006
2008
2010
2012
2014
Ingoing contact chain - Slaughter animal markets
0
100
200
300
400
500
600
700
2006
2008
2010
2012
2014
Outgoing contact chain - Slaughter animal markets
0.0
0.5
1.0
1.5
2.0
2006
2008
2010
2012
2014

Figure 6. In-going and out-going contact chains for miscellaneous sites.

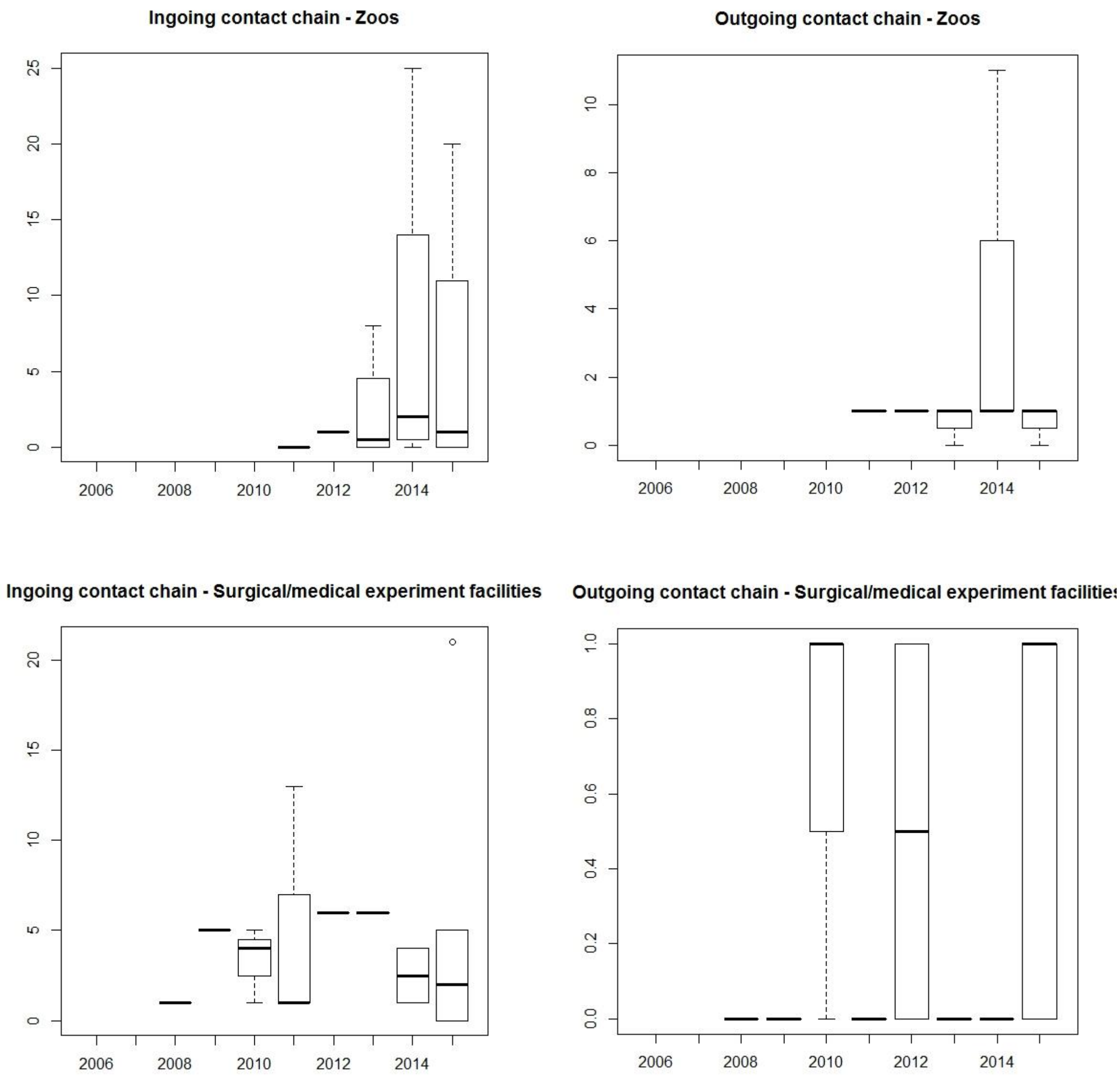

Figure 7. In-going and out-going contact chains for end of production sites.

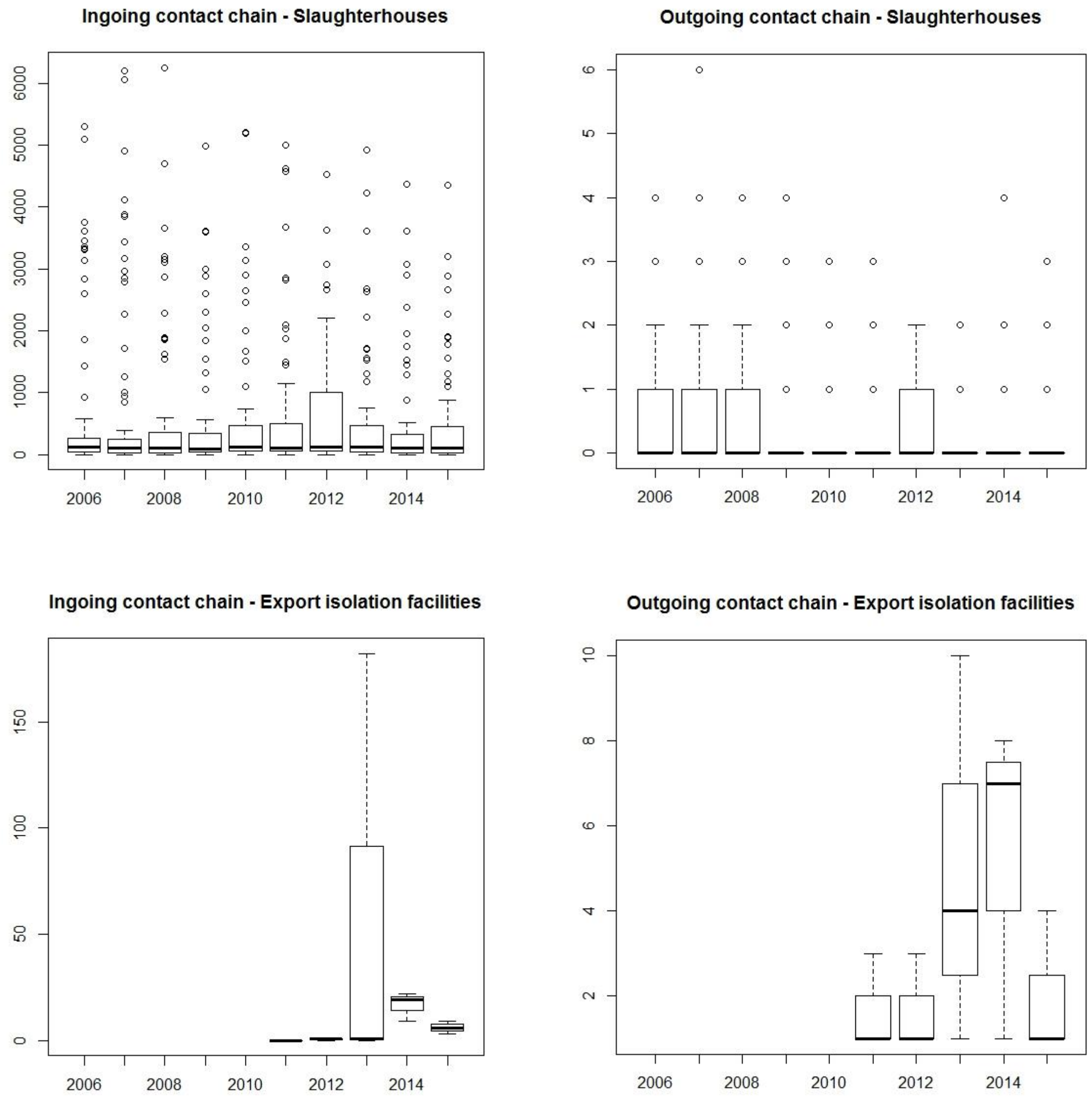

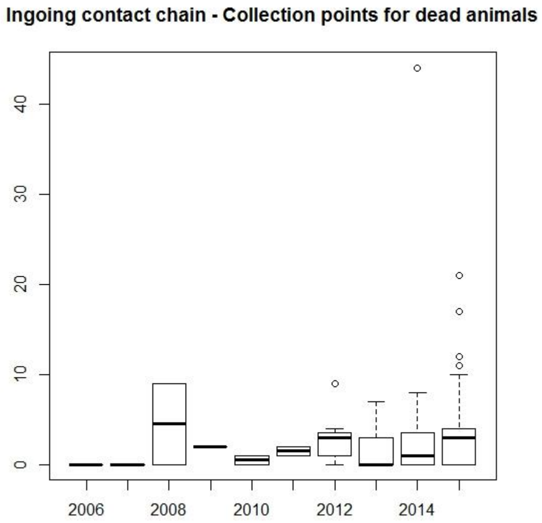
Ingoing contact chain - Collection points for dead animals
0
10
20
30
40
2006
2008
2010
2012
2014

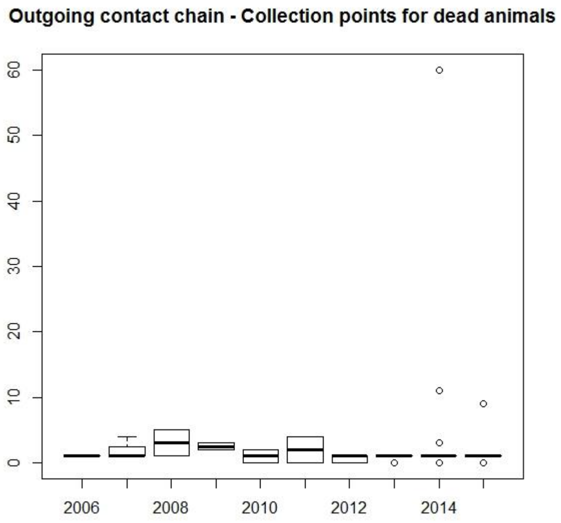
Outgoing contact chain - Collection points for dead animals
0
10
20
30
40
50
60
2006
2008
2010
2012
2014

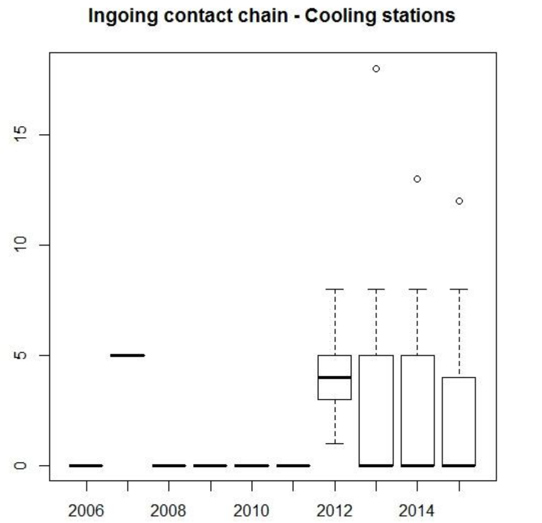
Ingoing contact chain - Cooling stations
0
5
10
15
2006
2008
2010
2012
2014

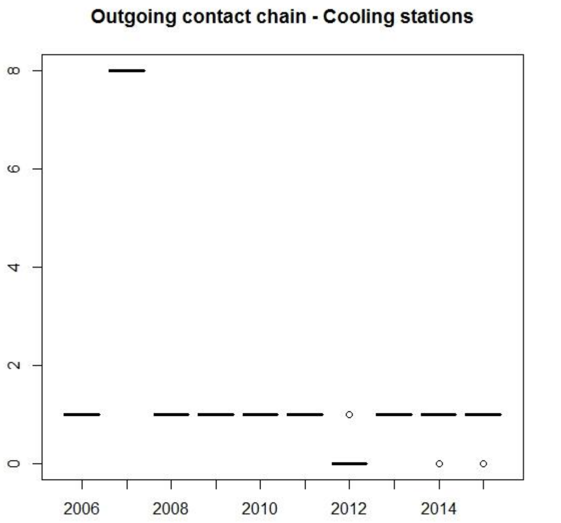
Outgoing contact chain - Cooling stations
0
2
4
6
8
2006
2008
2010
2012
2014

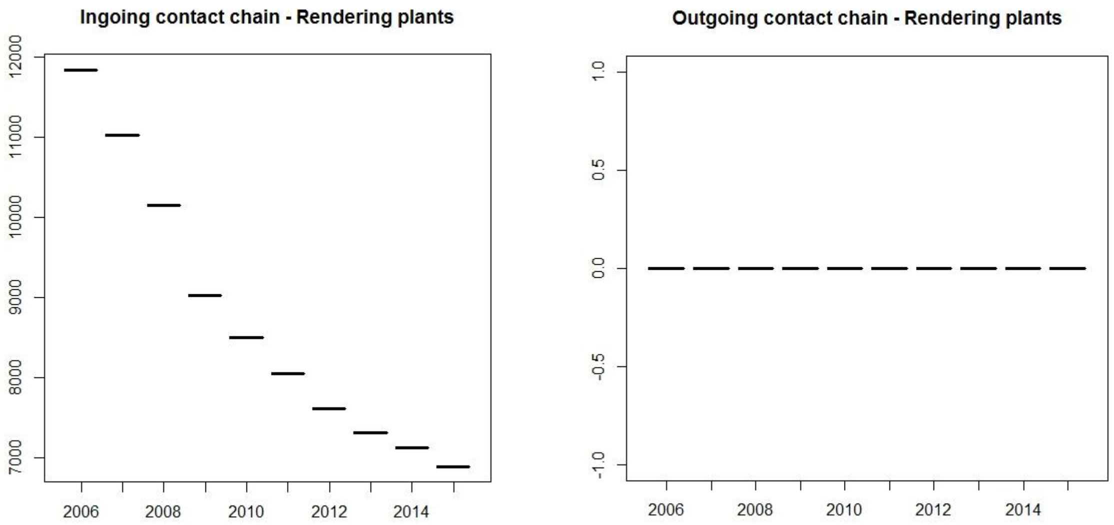
Ingoing contact chain - Rendering plants
12000
11000
10000
9000
8000
7000
2006
2008
2010
2012
2014
Outgoing contact chain - Rendering plants
1.0
0.5
0.0
-0.5
-1.0
2006
2008
2010
2012
2014
